# Nucleolar sub-compartments in motion during rRNA synthesis inhibition: Contraction of nucleolar condensed chromatin and gathering of fibrillar centers are concomitant

**DOI:** 10.1371/journal.pone.0187977

**Published:** 2017-11-30

**Authors:** Pavel Tchelidze, Aassif Benassarou, Hervé Kaplan, Marie-Françoise O’Donohue, Laurent Lucas, Christine Terryn, Levan Rusishvili, Giorgi Mosidze, Nathalie Lalun, Dominique Ploton

**Affiliations:** 1 Faculty of Exact and Life Sciences, Department of Morphology, Tbilisi State University, Tbilisi, Georgia; 2 EA 3804 (CRESTIC), Université de Reims Champagne Ardenne, Reims, France; 3 Université de Reims Champagne Ardenne, Reims, France; 4 Laboratoire de Biologie Moléculaire Eukaryote, Centre de Biologie Intégrative (CBI), Université de Toulouse, CNRS, UPS, Toulouse, France; 5 Platform of Cellular and Tissular Imaging (PICT), Université de Reims Champagne Ardenne, Reims, France; 6 CNRS UMR 7369, Université de Reims Champagne Ardenne, Reims, France; Universite Laval, CANADA

## Abstract

The nucleolus produces the large polycistronic transcript (47S precursor) containing the 18S, 5.8S and 28S rRNA sequences and hosts most of the nuclear steps of pre-rRNA processing. Among numerous components it contains condensed chromatin and active rRNA genes which adopt a more accessible conformation. For this reason, it is a paradigm of chromosome territory organization. Active rRNA genes are clustered within several fibrillar centers (FCs), in which they are maintained in an open configuration by Upstream Binding Factor (UBF) molecules. Here, we used the reproducible reorganization of nucleolar components induced by the inhibition of rRNA synthesis by Actinomycin D (AMD) to address the steps of the spatiotemporal reorganization of FCs and nucleolar condensed chromatin. To reach that goal, we used two complementary approaches: i) time-lapse confocal imaging of cells expressing one or several GFP-tagged proteins (fibrillarin, UBF, histone H2B) and ii) ultrastructural identification of nucleolar components involved in the reorganization. Data obtained by time lapse confocal microscopy were analyzed through detailed 3D imaging. This allowed us to demonstrate that AMD treatment induces no fusion and no change in the relative position of the different nucleoli contained in one nucleus. In contrast, for each nucleolus, we observed step by step gathering and fusion of both FCs and nucleolar condensed chromatin. To analyze the reorganization of FCs and condensed chromatin at a higher resolution, we performed correlative light and electron microscopy electron microscopy (CLEM) imaging of the same cells. We demonstrated that threads of intranucleolar condensed chromatin are localized in a complex 3D network of vacuoles. Upon AMD treatment, these structures coalesce before migrating toward the perinucleolar condensed chromatin, to which they finally fuse. During their migration, FCs, which are all linked to ICC, are pulled by the latter to gather as caps disposed at the periphery of nucleoli.

## Introduction

The nucleolus is a highly dynamic compartment inside the non-random 3D architecture of the genome, whose key function consists of ribosome biogenesis [1–10]. Microscopists discern the nucleolus together with surrounding condensed chromatin domains (or *perinucleolar compartment*) [11, 12] as the largest and densest nuclear compartment (S1 Fig). This structural and metabolic unit assembles in the course of post-mitotic unfolding of chromosomes into individual *Chromosomal Territories* (CTs) followed by genome reactivation [13–15]. Posed as a specialized chromosomal locus for ribosome synthesis, the nucleolus comprises the basic features of both *Chromatin Domains* (CDs) and chromatin-associated *Nuclear Bodies* (NBs). The nucleolus integrates the gene-rich CDs that consist of eukaryotic rDNA loops–the giant tandems built by hundreds of rRNA gene (r-gene) repeats with an uninterrupted head-to-tail arrangement. The non-nucleosomal open structure of transcriptionally competent rDNA chromatin (r-chromatin) unmasks the position of r-gene clusters in mitotic chromosomes that can be distinguished as discrete stretches termed *Nucleolus Organizing Regions* (NORs) [16–21]. Mammalian karyotypes mostly reveal several pairs of NOR-bearing chromosomes per diploid set. For example, there are 10 NORs detected in humans, all mapped to short arms of five acrocentric chromosomes pairs (N° 13, 14, 15, 21, 22) [3, 10, 22–24]. Only r-genes are clustered within NOR-bearing acrocentric chromosomes, being positioned between the telomere and centromere, adjacent to heterochromatic chromosomal segments. The rDNA arrays are flanked by sequences of heterochromatic nature, identified as the *Proximal Joint* (PJ, on the centromeric side) and the *Distal Joint* (DJ, on the telomeric side) [25, 26]. Being the largest chromatin-associated nuclear body [27–29], the nucleolar territory harbors an enormous number of r-gene expression products: the large 47S rRNA precursors assemble cotranscriptionally with ribosomal proteins and ribosomal assembly factors to form the 90S particles, which give rise to pre-40S and pre-60S particles at various stages of maturation upon endonucleolytic cleavages.

Nucleolar functions related to ribosome factories are properly organized within the confines of distinct sub-compartments defined as *Nucleolar Components* (NCs). These appear in light and transmission electron microscopes (LM and TEM, respectively) due to their unique structures, mediated by r-gene expression products and specific protein signatures [1–3, 30–34]. The pre-rRNA synthesis, processing and pre-ribosome assembling products are packaged around the r-chromatin transcription sites according to the sequence of the main steps of ribosome biogenesis. Transcription and processing factories are distributed within three basic ordered NCs giving rise to a tripartite nucleolar structure [4, 6] that is observed in TEM according to the appearance and density of the main NCs (S1B Fig). In a transcriptionally competent nucleolus, non-nucleosomal r-chromatin is shared among numerous *Fibrillar Centers* (FCs)–pale-stained NCs that have long been identified as an interphase counterpart of mitotic NORs. The two other NCs constitute the *Dense Fibrillar Component* (DFC) and a relatively opaque *Granular Component* (GC). The interface area between FC and the adjacent DFC is known as transcriptionally active r-genes territory [35]. The DFC and GC correspond respectively to early and late processing sub-compartments, where maturing 47S pre-rRNA molecules being cleaved, modified and assembled with ribosomal proteins, generate 40S and pre-60S particles containing the precursors to 18S and to 28S, 5.8S and 5S rRNAs, respectively [1–3, 6–10, 36].

Nucleolus-associated DNA (naDNA) domains presumably contain not only r-genes. In this respect, two additional chromatin-associated NCs with still no identified roles in nucleolar organization and functions are of particular interest. These are defined as members of nucleolar chromatin (so called *Nucleolus Associated Chromatin*, NAC) and appear in the form of a shell of perinucleolar condensed chromatin (PCC) that extends as strands of intranucleolar condensed chromatin (ICC). The latter are located into small interstices or expanded *Nucleolar Vacuoles* (NVs) that are non-membrane limited light zones in continuity with nucleoplasm. Preferential visualization of nucleolar chromatin domains on ultrathin sections shows that ICC and PCC are constituted of 10–30 nm thick nucleosomal fibrils and represent a single system passing through the interstitial network. Quite frequently FCs come in direct contact, and even can link to one another, with the ICC [37–40]. The significance of nucleolar chromatin in the spatial organization and/or regulation of nucleolar functions as well as the mechanisms of its origin are still obscure.

The nucleolus arises at the end of telophase due to the synchronous reactivation of rRNA synthesis on rDNA loops extending from several NORs into the developing nucleolar space. During nucleologenesis, interphase NORs reorganize into rDNA-containing CDs (rCDs) resulting from spatial interaction between non-randomly compartimentalized CTs, perinucleolar centromeric and telomeric heterochromatin, as well as non-nucleolar genomic regions. Being a derivate of interphase NOR-bearing CTs, the nucleolus seems to be reassembled in compliance with the functional topography of nucleolus-associated chromosomal segments, including non-ribosomal sequences of telomere and centromere satellite DNA. The bulk of data, including quite recent results, indicates the particular role of intra- and perinucleolar heterochromatin in nucleolar stability. Despite their non-ribosomal origin, rDNA flanking regions, the centromeric and telomeric heterochromatic segments together with non-coding RNAs (*Ribosomal Intergenic Spacer Long Noncoding RNA*, *IGS lnc RNA)* may be crucial for postmitotic establishment and maintenance of the global nucleolar structure organized in the form of functional sub-compartments [20, 21, 26, 41–51]. Whether rDNA-adjacent heterochromatic chromosomal segments determine the exclusive position of the nucleolus within the territorial architecture of the nucleus or contribute to the nucleolar organization and stability via structural and functional interactions with surrounding nucleolar chromatin remains to be elucidated.

In the present work we studied the 3D organization of FCs and of NAC to address whether these structures constitute parts of a same entity. The best model for such studies is nucleolar inactivation in response to the cessation of rRNA synthesis, causing emergence of a few giant FCs associated with prominent zones of DFC and large ICC clumps. As an inhibitor of rRNA synthesis, Actinomycin D (AMD) induces a typical spatial reorganization of the nucleolar structure that has long been known as segregation of nucleolar components. Nucleolar segregation can be easily determined by phase contrast microscopy by light and dark “caps” that correspond in fact to FCs and DFCs reshaped into large crescent-like structures that appear to be pressed onto the surface of round nucleolar remnants [2, 22, 52–55]. Our hypothesis is that the dynamics of nucleolar components during nucleolar segregation and capping may be caused by a concerted gathering of nucleolar associated chromatin pulling FCs and DFC to the perinucleolar condensed chromatin shell. To study this phenomenon, we combined 4D imaging of FC and of nucleolar chromatin in living cells with TEM structural studies of the nucleolus within the same cells. To reach this goal, we developed a new technical approach that correlates fluorescent live-cell 3D imaging with postfixation immunolabeling and high resolution EM analysis of nucleolar organization performed in the same cell. For this, images of the nucleolar ultrastructure were merged with confocal microscopy (hereafter CM or LSM) images indicating the spatial distribution of UBF, a specific protein marker of uncondensed r-DNA genes, while the nucleosomal ICC was identified by histone H2B-GFP fluorescence. This strategy allowed us to study in great detail the spatial arrangement of nucleolar chromatin and its relationship with other nucleolar compartments as well as the mechanisms involved in the displacement of FCs from the nucleolar interior to its periphery during rRNA synthesis inhibition.

## Materials and methods

Time-lapse confocal microscopy imaging of fluorescently tagged single living cells imaged by correlative light and electron microscopic analysis (CLEM) was performed on HeLa cells stably expressing histone H2B-GFP (courtesy of Dr K. Monier, ENS Lyon, France) or fibrillarin-GFP (courtesy of Dr. P. Roussel, Pierre and Marie Curie University, Paris, France). We developed our correlative study as follows. Initially, we followed the spatial organization and dynamics of the rearrangement of FC by 3D imaging and time-lapse confocal microscopy, using both fixed and living KB cells expressing or co-expressing fibrillarin-GFP, UBF-GFP/dsRed, and histone H2B-GFP fusion proteins as well as HeLa cells stably transfected with fibrillarin-GFP and histone H2B-GFP. In the second step, the reorganization of nucleolar chromatin induced by AMD was studied by 4D CM in HeLa cells expressing histone H2B-GFP. At different time points cells were fixed, immunolabeled for UBF, imaged for simultaneous localization of intra-nucleolar condensed chromatin and FCs, and finally processed for TEM analysis. By using a precise positioning and orientation system, we were able to find exactly the same cells and image them by EM and then to subject the chosen cells to the following Confocal/TEM Overlay (CTO) [56].

### Cell culture

HeLa and KB cells were used because they show prominent intranucleolar histone H2B-GFP fluorescence (S1C and S2 Figs) corresponding to the nucleosomal domains with the ultrastructural appearance of ICC. In the experiments dedicated to LM/EM distribution of UBF, fibrillarin, and histone H2B in living and fixed cells we applied transient transfection using human KB cells. KB cells contain well visible intra-nucleolar inclusions of ICC (S2 Fig) and large FCs associated with prominent DFC zones that facilitate LM discrimination of nucleolar sub-territories occupied by r-gene transcription and pre-rRNA processing machineries (S1A and S1B Fig). In addition, preliminary experiments revealed high stability of KB cells to damage provoked by the transfection procedure that yielded a large quantity of mono- and doubly-transfected cells.

Cultures were either mono-transfected with UBF-GFP or fibrillarin-GFP plasmids or co-transfected with H2B-GFP and UBF-dsRed plasmids. Transfected and non-transfected stock cultures were maintained in 40 ml flasks containing DMEM (Gibco, UK) supplemented with 10% calf serum and 1% penicillin/streptomycin mixture. Cells were reseeded in new medium 2–3 times per week, as soon as the monolayer became late preconfluent or early confluent. Tests for mycoplasma detection were performed monthly.

For inhibition of rRNA synthesis, cells were treated during 1–8 h by a low dose (0.05 μg/ml) of AMD (Sigma, USA) in two different conditions: 1) Working with a Bio-Rad MRC-1024ES Laser confocal microscope (LCM), we delivered the drug using a Bioptech (USA) perfusion system (for details see below). We noted that in this condition, the AMD concentration did not reach a high level immediately and thus, nucleolar remodeling developed slowly and took around 7 h to reach complete segregation. 2) Therefore, working with a Zeiss 710 NLO LCM and ILAS Spinning Disk confocal system (SDCS) we delivered AMD by direct addition to the culture medium for immediate contact of cells with the full concentration of drug; in these conditions nucleolar remodeling developed more rapidly so that we reached complete segregation after around 2–3 h. The same procedure was used for UBF fluorescence studies in fixed cells.

### Transient transfection, observation and imaging of KB cells

A suspension of 5 x 10^5^ KB cells/ml was seeded onto the surface of Ø40 mm glass coverslips (Bioptech) placed in a Ø50 mm plastic Petri dish. Fugene-6 (Roche Diagnostics, Switzerland) was used to transfect cells with a cDNA construct coding for UBF-GFP, fibrillarin-GFP or histone H2B-GFP or in some experiments with two plasmids coding for H2B-GFP and UBF-dsRed. Coverslips were fixed in 4% paraformaldehyde (PAF, EMS, USA), mounted in Citifluor AF1 (Agar Scientific, UK) and stored at 4°C. Alternatively, part of the transfected and fixed cells was submitted to anti-GFP immunolabeling and TEM analysis. Preparations selected for LCM were examined and imaged on a Bio-Rad LCM (described above) combined with an Olympus IX70 fluorescence microscope using a Plan-Apochromat/x60/1.40/Oil objective (for details see S1–S3 Methods).

### Conventional TEM analysis

Sub-confluent KB/UBF-GFP and HeLa/H2B-GFP expressing cells on Ø40 mm coverslips were rinsed (3 x 5 min) with 0.1M phosphate buffer (Biomérieux, France) and then fixed during 10 min with 4% PAF and 2.5% glutaraldehyde (both from EMS) in 0.1M phosphate buffer. After postfixation in 1% OsO4 (EMS) in the same buffer, the cells were rinsed with phosphate buffer and distilled water (MiliQ, Millipore, USA), scraped from coverslips, sedimented in 30% BSA (Sigma-Aldrich, USA) using 0.5 ml centrifuge tubes, and jellified after removing BSA by adding to the cell pellet a few drops of 25% glutaraldehyde. The pellets were cut into ~1mm^3^ pieces, washed in distilled water overnight, and then dehydrated in a series of acetone (Sigma, USA) in deionized water up to 100% acetone. Then cells were infiltrated in a mixture of 100% acetone-Embed 812 (EMS) 1:1 and 1:2 (overnight). The impregnation was continued with 2 changes of pure Embed 812. Finally, the samples were placed for polymerization at 60°C in BEAM capsules (#3, EMS) filled with a fresh portion of Embed 812. Silver-gold sections (~0.1 μm) cut using a Reichert-Young Ultracut ultramicrotome (Reichert, Austria) were contrasted with 5% aqueous uranyl acetate and lead citrate (both from Merck, USA) and then observed and imaged in a Hitachi H300 electron microscope at 70 kV.

### Pre-embedding anti-GFP immuno-TEM labeling of UBF-GFP transfected KB cells

Ultrastructural localization of GFP-tagged proteins in fixed transfected KB cells was performed by using anti-GFP immuno-TEM, combining nanogold (Nanoprobes, USA) labeling with silver enhancement (HQ Silver Kit, Nanoprobes, USA) [57]. To detect GFP-positive sites in TEM we used the following antibodies: (i) anti-human GFP mouse monoclonal (Roche Diagnostics); (ii) goat anti-mouse (Jackson, USA); (iii) streptavidin-nanogold conjugate (Nanoprobes). Cells were harvested without osmication by meticulous scraping from the glass surface, then dehydrated and embedded in epoxy resin as described in the previous section. Ultrathin sections were picked up on 200 mesh copper grids (EMS) and stained with uranyl acetate and lead citrate and examined and imaged as described above (for details see S4 Method).

### Time-lapse observation and imaging of living KB cells

To trace the dynamics of fibrillarin and UBF redistribution in the course of nucleolar segregation during AMD treatment we performed long-term 4D studies using fibrillarin-GFP and UBF-GFP expressing cells growing on Ø40 mm coverslips, using a living cells observation system based on a Bioptechs FCS2 incubation chamber with a specimen and objective temperature control module and peristaltic pumps that enabled perfusion with fresh medium and delivery of AMD at 37±0.1°C. The flow of the pumps was adjusted to 10 ml/h. Images were collected using a Bio-Rad MRC-1024ES/Olympus IX70 LCM in fast scanning mode (1/2 sec for each 512x512 image) to diminish the exposure of cells to the laser beam. To demonstrate the general features of the behavior of components containing fibrillarin and UBF during nucleolar segregation we used simple visualization tools in the form of 2D movies (standard 2D+time mode) provided by ImageJ or QuickTime. For spatial reconstruction with subsequent dynamic modeling in the form of 4D movies we utilized a more sophisticated approach, Rev4D software developed by the CRESTIC group (University of Reims, Reims, France) to analyze and visualize the evolution of multiple topology-changing structures during their deformation, division, fusion, etc. [58, 59]. To demonstrate redistribution of nucleolar proteins in 4D the most significant data were extracted from time/z-series, then 3D reconstructed using Amira 5.5 software (TGS, Mercury Computer Systems, France) and finally displayed in chronological order as a gallery of consequential volume models (for details see S5 Method).

### Imaging of histone H2B and UBF in living HeLa cells

To study the 4D dynamics of the ICC and its relationship with FCs during nucleolar segregation and capping, we used a CLEM approach combined with post-fixation immunolabeling of UBF. For time-lapse imaging a suspension of histone H2B-GFP tagged HeLa cells at 60–70% confluence was plated on Ø35 mm uncoated glass “MatTek” or “Ibidi μ-Dish-500” Petri dishes (MatTek, USA and Ibidi GmbH, Germany) with Ø14 mm and Ø21 mm wells as growth area. The bottom coverslip of these dishes is an etched finder grid that facilitates the search for regions/cells of interest (ROI/COI). After 24–48 h of incubation the cells were briefly rinsed 3x with PBS and incubated in fresh medium at 37°C for 2–3 h before experiments.

All procedures connected with confocal microscopy such as selection and marking of appropriate cells or groups, capture of control z-stacks as well as time-lapse imaging during AMD action, were conducted at 37°C in a CO_2_-enriched atmosphere using a special microscope plate holder with a close-fitting lead. To outline a ROI, the culture was first previewed and recorded under low magnification on a Zeiss 710 NLO LCM and Spinning Disk ILAS2 confocal system (SDCS) (Roper Scientific, USA) coupled with a Zeiss Axioobserver inverted microscope with a 512x512 EMCCD camera (for details see S6 Method).

### Postfixation imaging of HeLa cells after anti-UBF immunolabeling

Imaging of labelled UBF is widely used to identify undercondensed active or inactive rDNA genes folded into the structure of interphase FC and metaphase NORs [15, 60–62]. To perform UBF labeling, AMD treated H2B-GFP transfected cells were immediately fixed at the end of time-lapse imaging. To label UBF we used: (i) mouse anti-UBF/F9-fragment monoclonal antibodies (Santa Cruz Biotechnology, USA); (ii) biotinylated goat anti-mouse antibodies (Jackson, USA); (iii) streptavidin-Alexa568 (Invitrogen Molecular Probes, USA). After immunolabeling, COI previously imaged during time-lapse experiments were found by their position on the finder grid. After 3D imaging of immunostained UBF, cells were prepared for TEM and CLEM analysis (for details see S7 Method).

### CLEM technique

Cells growing in MatTek dishes were fixed in 4% PAF, rinsed in PBS, and post-fixed in 2.5% glutaraldehyde and 1% OsO4 in 0.1M phosphate buffer (Biomérieux) and rinsed with PBS and distilled water. The cells were dehydrated through a series of ethanol-deionized water mixtures up to 100% ethanol, infiltrated in a mixture of 100% ethanol-Embed 812 (EMS) 1:1 and 1:2, and then with pure Embed 812, and polymerized in dishes at 60°C. Silver-gold serial ultrathin sections (~0.1μm) were picked up using coated one-hole copper slot grids (Pella, USA) or uncoated H7 Hexagonal Maxtaform grids (EMS), stained with uranyl acetate and lead citrate, and imaged by electron microscopy as described above (for details see S8 Method).

### CM/TEM overlay of doubly-tagged HeLa cells

To merge identical ROI imaged by CM and EM within the same cell, a modified CTO technique was utilized [56]. For merging of the two images it is essential that the ultrathin and optical sections are at approximately the same depth and orientation of a given nucleus/nucleolus. Thus we used serial ultrathin sectioning; the block face, trimmed for ultramicrotomy, was adjusted relative to the knife so that every point of the surface containing the COI was at the same distance from the knife edge. Further sectioning provided sections beginning at the top surface of the block and penetrating the cell in the Z direction to a depth of several tens of μM. Thus the appropriate plane of interest containing nucleoli could be chosen during examination of serial sections in TEM and its depth was noted. Then the corresponding depth on LSM image stacks was calculated and appropriate planes were visualized using virtual serial slicing. Overlays were performed using Corel Draw software. Serial fluorescent and EM images were adjusted to the same magnification and nuclei/nucleoli, FCs, ICCs, and NVs were contoured in different colors on transparent plastic sheets using fine permanent markers, overlaid, and roughly aligned with the TEM image below and the confocal image above. The transparency of the confocal image was adjusted so that the TEM image was seen and the images were precisely aligned by adjusting their orientation and magnification.

A total of 21 different experiments were performed. In each of them, a minimum of 10 cells were observed and different ROI were imaged. For example, although ROI shown on S9 Fig contains 9 interesting cells, only 4 cells from this series (marked by numbers) were used for CLEM. Altogether, twenty double-labeled cells belonging to different experimental series were fully analyzed using the CLEM approach. In order to display nucleolar changes accompanying the gradual decrease in number of FCs, we show the three most demonstrative cases that appeared after treatment with AMD for 1 h. These were: cells N°1 and N°2 containing pre-segregated nucleoli with several FCs from series shown on S9 and S14A–S14E Figs and one cell from another series, containing one ring-shaped nucleolus with one FC (S14F–S14I Fig). Additionally, two nucleoli were analyzed in cell N°2.

## Results

### Nucleoli remain independent units upon inhibition of rRNA synthesis

**Fig 1:**
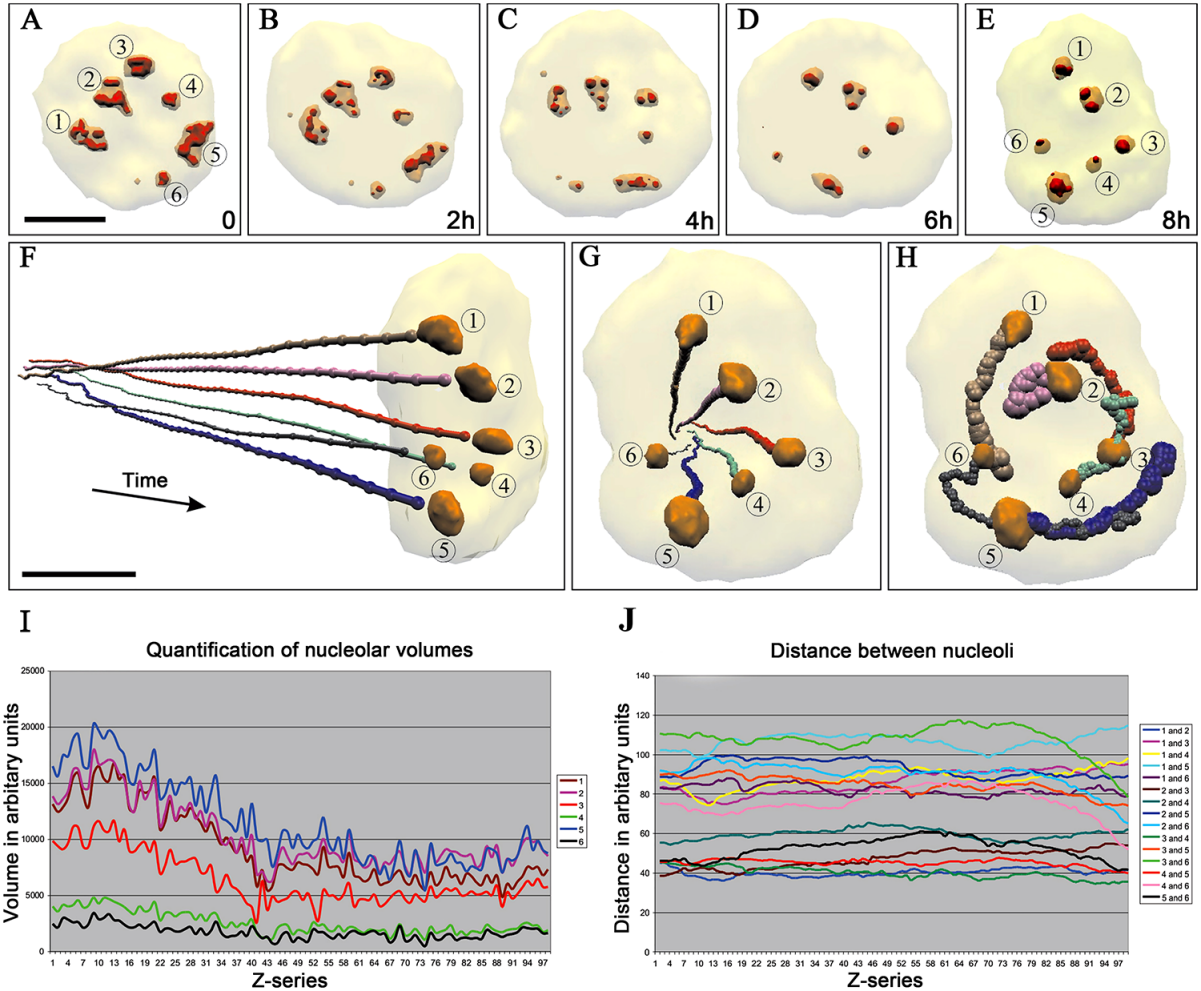
4D evolution of nucleolar volume and topography during AMD treatment in living KB cells. One hundred Z-stacks were acquired during 8 h and images were generated by Rev4D software. (A–H) Gallery of 3D reconstructions displaying nuclear and nucleolar evolution and showing the whole nucleus (low threshold, transparent yellow), the nucleoli (medium threshold, transparent orange) and the DFC (high threshold, solid red) (same cell as in S8 Fig and S1–S5 Movies). (F–H) Trajectories of the center of mass of each nucleolus, demonstrating that their relative position is fixed, that there is no nucleolar fusion, and that all nucleoli rotate around nucleolus #2 (see traces of nucleoli 1 to 6 on H). The scale bars represent 10 μm in (A); 8 μm in (F). (I, J) Changes of volume and topography of six nucleoli within the same nucleus, shown in different colors. During inhibition, nucleoli decrease in size (I) and are positioned at a constant distance from each other (J).

Time-lapse imaging of cells containing fibrillarin-GFP was performed to study the 3D dynamics of nucleoli during inhibition of rRNA synthesis by AMD. Fibrillarin was chosen because: i) it is present at high concentration within the DFC where its rRNA methyl transferase activity is required for rRNA processing, and ii) it is also present but at lower concentration within the other nucleolar compartments and within the nucleoplasm. These characteristics allowed the simultaneous imaging of DFC, whole nucleoli, and nucleoplasm and the study of their 3D modifications during time series. Thus, 100 *z*-stacks containing 60 optical sections each were acquired every 5 min for 8 h. Projection of the sections in each z-stack was performed to create a movie (S1 Movie) showing the modifications arising in a representative cell, so that we were able to simultaneously visualize: i) the reorganization of DFC within each nucleolus, ii) all nucleoli, and iii) the contour of the nucleus. Using surface visualization we observed that each nucleolus, which was initially extended and irregularly shaped, became spherical during inhibition of rRNA synthesis (for example, nucleolus N°2 in Fig 1, S3 Fig and S1 and S2 Movies; see Fig 1 for numbering).

Using Rev4D software, we simultaneously visualized the 3D organization of DFC (high threshold, solid red), of nucleoli (medium threshold, transparent orange) and of whole nuclei (low threshold, transparent) yellow (Fig 1). To analyze the behavior of nucleoli, we identified their center of mass at each time-point and calculated their trajectories in space and time, their relative distance, and their volume. These analyses showed that during AMD treatment, nucleoli maintained their relative distance although they rotated by around 90° in the nucleus (the center of rotation is close to nucleolus N°2) and they lost half of their initial volume (S3–S5 Movies).

Altogether, these data demonstrate that inhibition of rRNA synthesis induces a strong internal reorganization of nucleoli (and of the DFC in different nucleoli) but never induces their fusion. Moreover, they show that nucleoli keep constant positions relative to one another during this process.

This analysis also showed that the re-organization of the DFC (S5 Movie) was a sequential process during which its cord-like structure was first broken into several fragments. Then, these fragments became spherical, came close together, and finally fused to constitute 1 to 3 caps at the border of each reorganized spherical nucleolus. As the DFC is the site of early steps of rRNA processing, we can hypothesize that its spatiotemporal behavior could be different from that of rDNA genes and of chromatin. Thus, in the following we investigated how active rDNA genes and condensed chromatin reorganize when rRNA transcription is inhibited by AMD.

### Inhibition of rRNA synthesis induces enlargement of nucleolar structures containing UBF

As UBF is specifically associated with active undercondensed rDNA genes [17–19] we used confocal microscopy of UBF-GFP within fixed KB cells to analyze the behavior of active rDNA genes (S4 Fig). In control cells (S4A–S4C Fig), GFP-UBF was present in numerous nucleolar spheroidal foci ~ 0.3–0.5 μm in diameter, disposed as so-called “necklaces” by 3D visualization. After 1 h with AMD (S4D–S4F Fig), all nucleoli became smaller and ovoid. They contained several prominent UBF-GFP positive spheres ~ 0.5–1 μm in diameter located within areas of low phase contrast. After 2 h of inhibition (S4G–S4I Fig), UBF-GFP fluorescence was present within 1–3 cap-like structures ~ 1–2 μm in their larger axis located on the outer part of spheroidal nucleoli within large caps of low phase contrast.

### Time-lapse confocal microscopy shows a sequential gathering and fusion of nucleolar structures containing UBF during inhibition of rRNA synthesis

**Fig 2:**
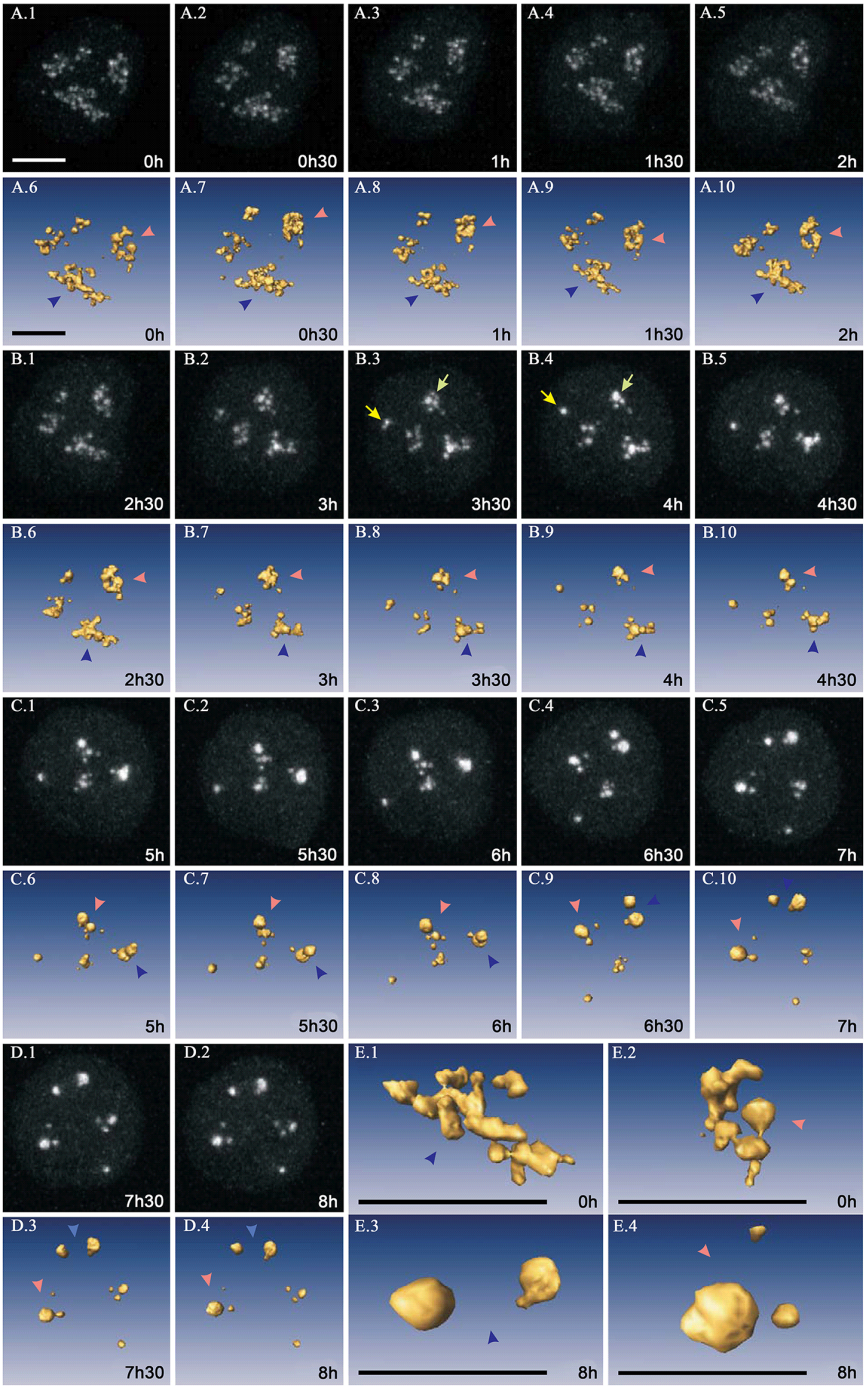
4D dynamics of UBF-GFP during inhibition of rRNA synthesis in living KB cells. (A–D), gallery of fluorescence maximum intensity projections and 3D reconstructions displaying the dynamics of UBF-GFP during nucleolar segregation. Reorganization of UBF-GFP spots was based on their successive gathering, fusion, enlargement, and final grouping into two or three caps localized at the nucleolar periphery. Blue and red arrowheads identify two large nucleoli to demonstrate their rotation within the nucleus during the step-by-step reorganization of UBF-GFP spots and (E1-E4) their 3D visualization at times 0 and 8 h respectively. The scale bars represent 10 μm.

To understand exactly how structures containing UBF are reorganized during AMD treatment, we studied living cells by time-lapse confocal microscopy. Thus, 100 *z*-stacks containing 60 optical sections each were acquired every 5 min for 8 h. For each *z*-stack, a projection of optical sections and 3D visualization were performed (for example Fig 2 and S6 Movie). Before AMD treatment, UBF-GFP fluorescence appeared as numerous spherical entities ~0.3–0.5 μm in diameter disposed as a 3D chain-like structure within 4 nucleoli (Panels A1 and A6 in Fig 2). From 30 min to 2.5 h of AMD treatment, all fluorescent UBF spots came close to each other but maintained their number and size.

Between 2.5 and 5 h, UBF-positive spots strongly reorganized within smaller and roundish nucleoli. Their number decreased due to their coalescence, whereas their individual size and fluorescence intensity increased markedly. Four to five hours after beginning AMD treatment, nucleoli contained a few large and brightly fluorescent aggregates which shifted toward the nucleolar periphery and fused to constitute two to three brightly fluorescent caps 0.7–3.5 μm in size. At 8 h, the four nucleoli contained UBF caps

**Fig 3:**
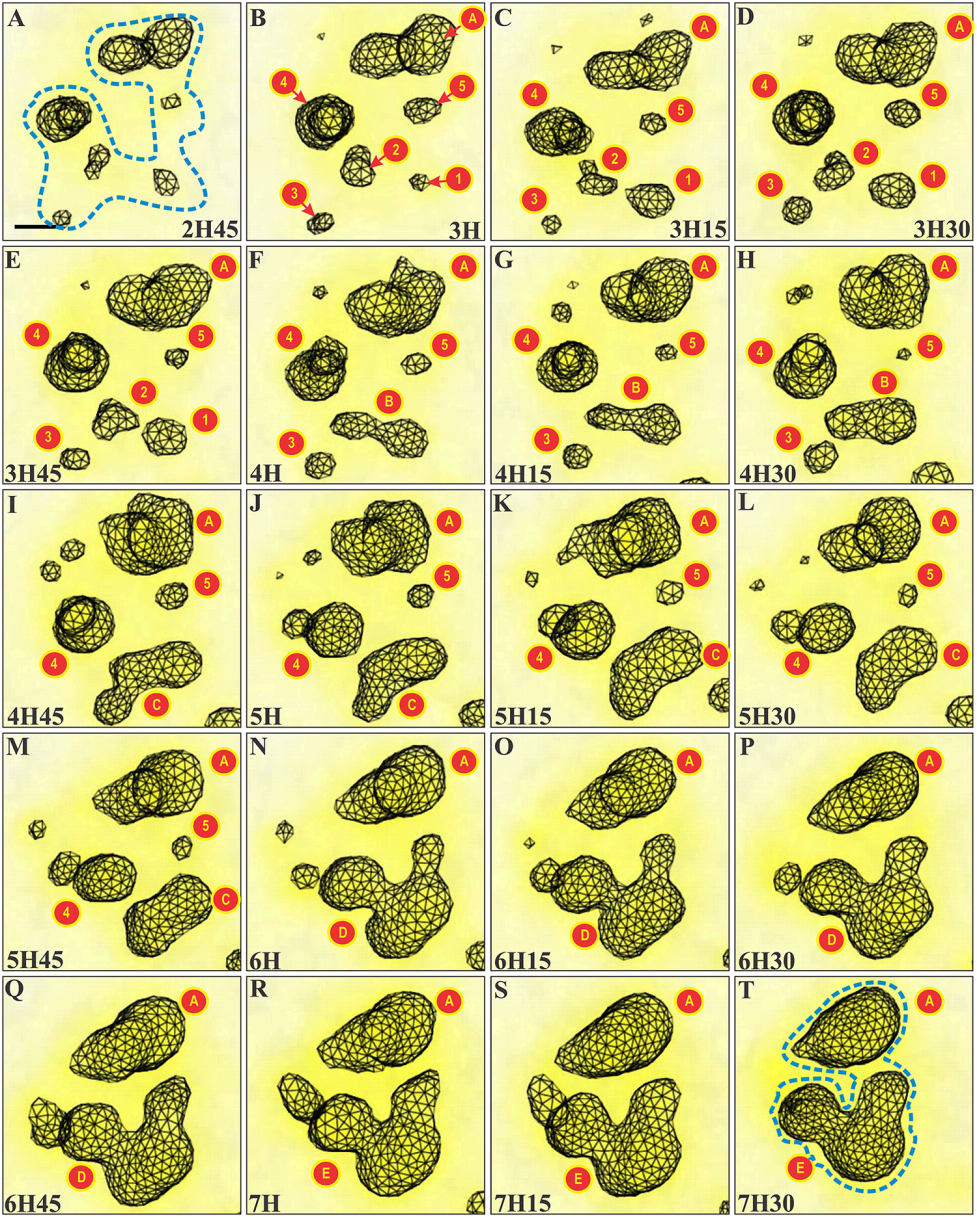
4D visualization of UBF-GFP dynamics during inhibition of rRNA synthesis in the same living KB cells as presented on Fig 2. (A–T) An example of 4D dynamics demonstrating successive fusion and gathering of five UBF positive spheroids (N°1 –N°5) contained in a ROI (delimited by the blue dotted line). Between 2 h 45 min and 7 h 30 min, we noticed the following steps: spheroids N°1and N°2 fused to give structure A; the latter gathered with N°3 to give B, and N°4 and N°5 finally gathered to B to give C. Note that the cap resulting from the fusion and gathering of the different spheroids is more compact than the initial region limited by the blue dotted line. The scale bar represents 2 μm.

To better analyze the step-by-step coalescence of UBF-GFP structures, we chose to zoom and orientate 5 of these structures during the period between 2 h 45 min and 7 h 30 min (Fig 3). Here, five UBF-positive spheroids (N°1—N°5) contained in a ROI (delimited by the blue dotted line) successively gathered to form a typical cap. Between 2 h 45 min and 7 h 30 min, spheroids N°1and N°2 fused to give structure A; the latter gathered with N°3 to give B and, finally, N°4 and N°5 gathered with B to give C. At the end of this process, C had a typical 3D cap structure which resulted from both fusion and gathering of the different spheroids and which was much more compact than the initial ROI.

Altogether, as UBF-GFP is a marker of under-condensed rRNA genes, we conclude that during AMD treatment fusion of rRNA gene clusters is restricted to each nucleolus and never occurs between clusters located in different nucleoli. To further question if condensed nucleolar chromatin behaves in the same way, we investigated its 3D reorganization by confocal microscopy in fixed and in living cells.

### The intra- and perinucleolar condensed chromatin network reorganizes strongly during AMD treatment

#### 3D organization of the ICC network in control HeLa cells

**Fig 4:**
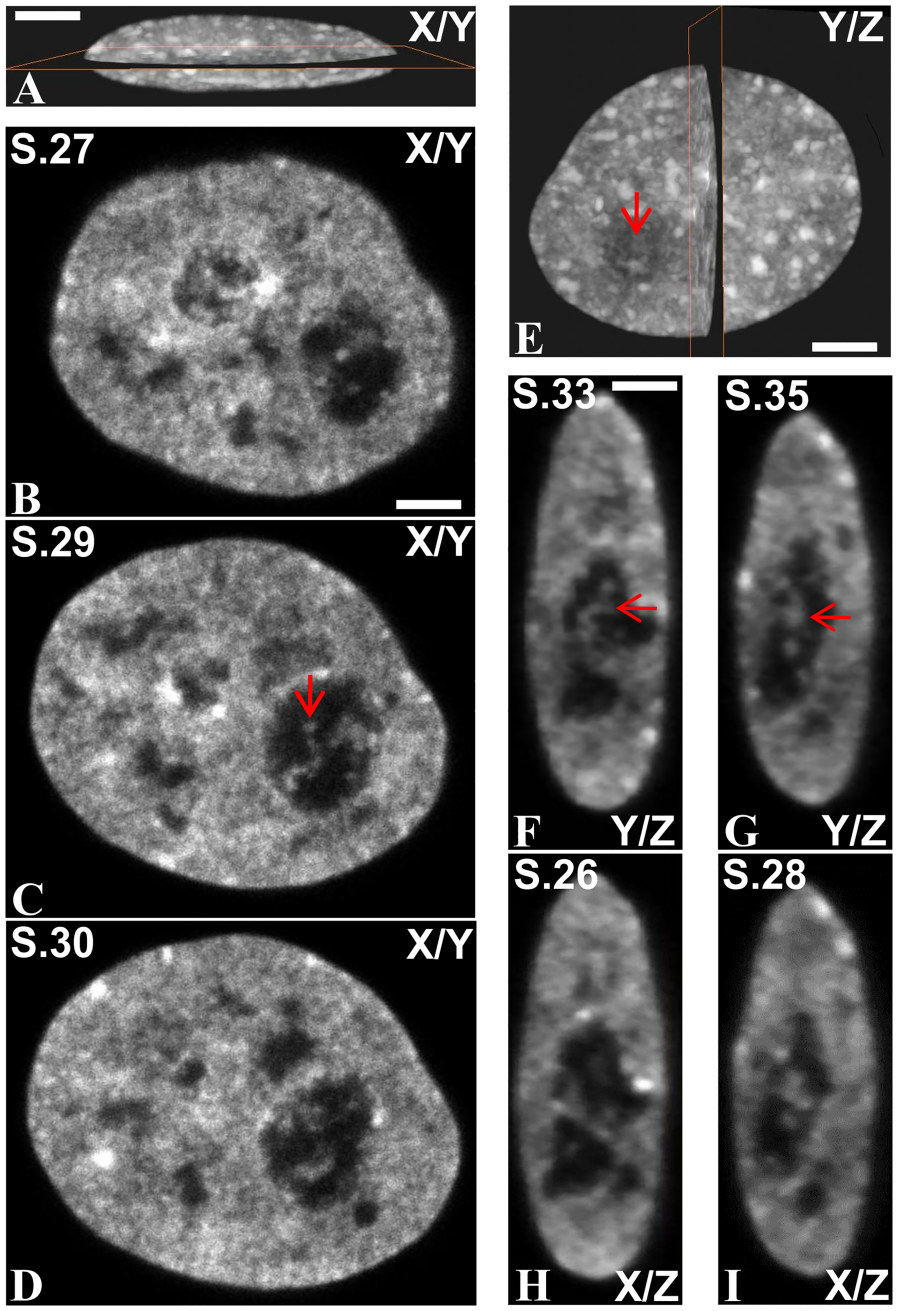
ICC network inside the nucleolar volume of one control HeLa cell expressing histone H2B-GFP. (A) X/Y plane section of the nucleus. (B–D) Three consecutive optical sections (sections 27 (S27), 29 (S29) and 30 (S30) passing through the nucleolus. The ICC network was clearly seen (red arrow on C). (E) Y/Z plane section of the nucleus. (F-I) Two consecutive Y/Z (F, G) and X/Z (H, I) plane sections at higher magnification. Red arrows indicate strands of ICC localized in the depth of the nucleolar volume. The scale bars represent 7 μm in (A); 4.5 μm in (B-D); 5.5 μm in (E); 4.5 μm in (F-I).

In fixed control KB and HeLa cells transiently or stably expressing histone H2B-GFP, we imaged a 3D network of nucleolar chromatin (Fig 4, S1C and S2 Figs) constituted of a shell of PCC in continuity with strands of ICC.

**Fig 5:**
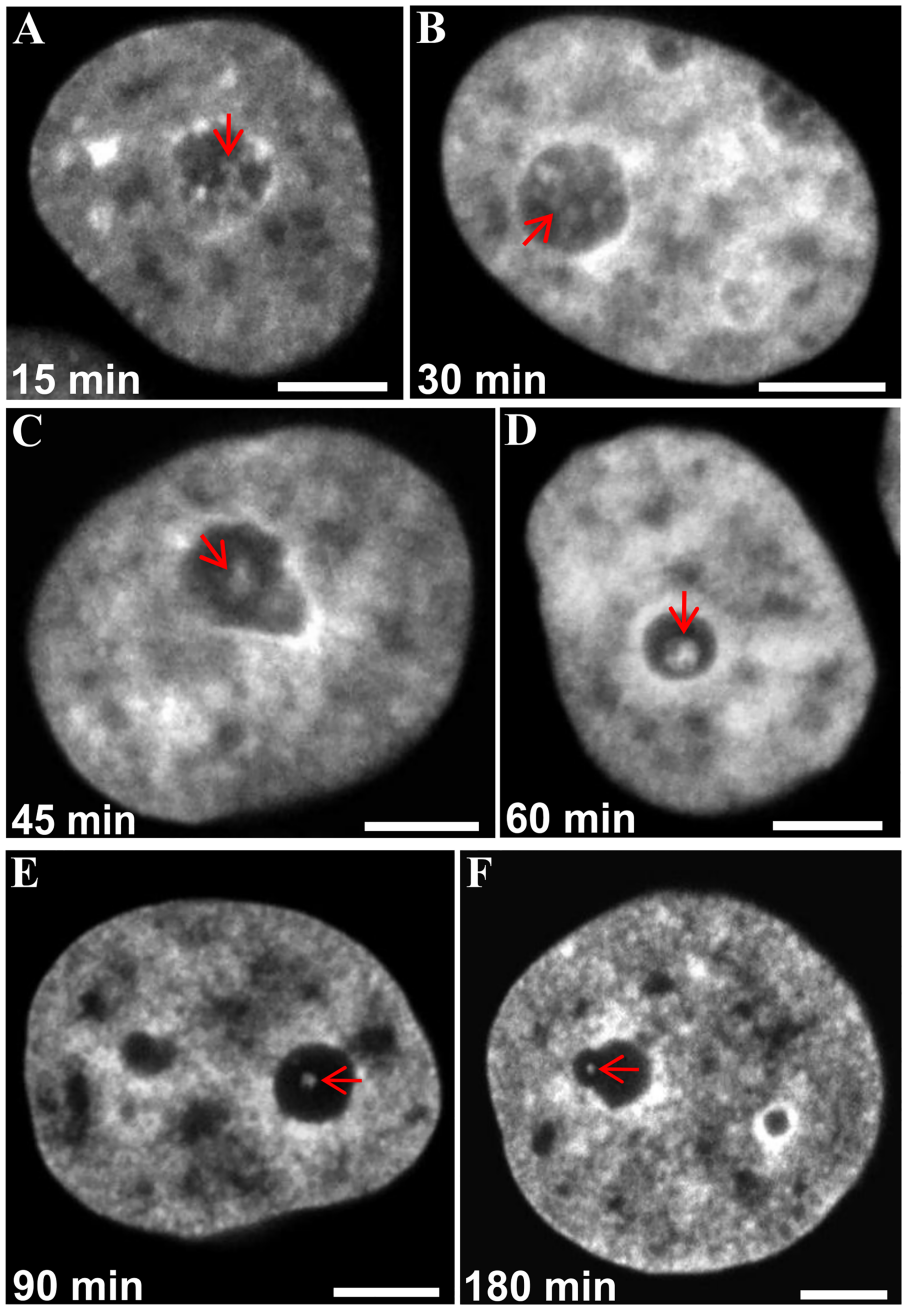
AMD-induced remodeling of ICC as revealed in fixed HeLa cells stably expressing H2B-GFP. (A–E) Gradual transition of ICC from a network-like organization (red arrows) to coarse clumps during 1.5 h of AMD treatment. (A) After 15 min the ICC network was more prominent than in control cells. (B) After 30 min the ICC network began coarsening. (C) After 45 min gradual shrinkage of the ICC network and fusion of individual ICC areas into large clumps became obvious. (D, E) During the next 45 min, ICC areas transformed into single large spheroids, often in contact with the PCC shell. (F) Nucleolar capping after 3 h; only tiny ICC inclusions (red arrows) attached to the inner margin of the nucleolar cap were observed. The scale bars indicate 6 μm in (A, B); 5 μm in (C); 4.5 μm in (D); 4 μm in (E); 4.5 μm in (F).

In cells fixed at different times during the action of AMD, we observed that the ICC network became less and less extended compared to control cells (Fig 5A–5E). After 3 h of treatment (I), the ICC was seen as a small single nucleolar spheroid which was often in contact with the inner surface of the nucleolar cap (Fig 5F) or PCC.

#### 4D dynamics of ICC and PCC during AMD treatment

**Fig 6:**
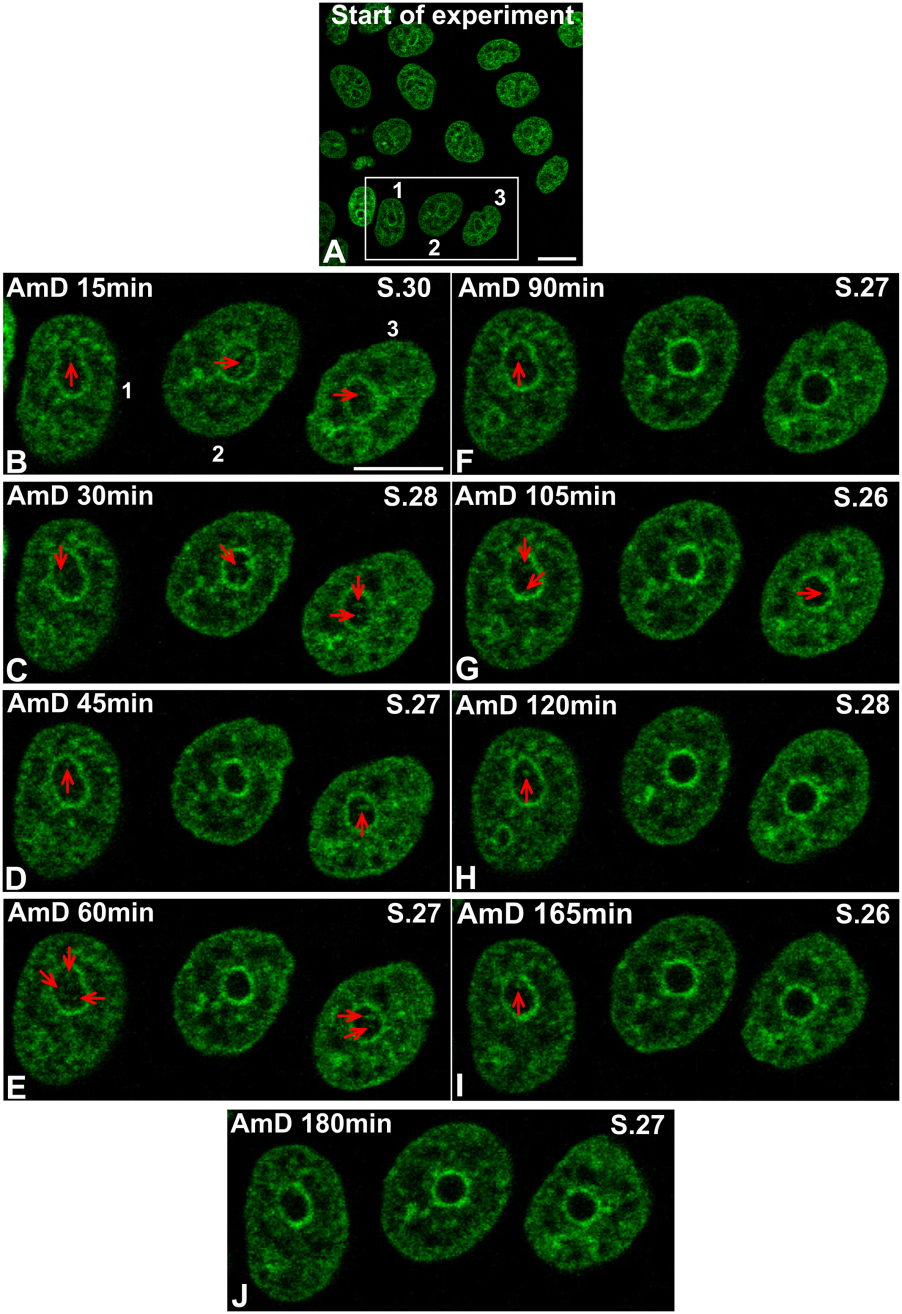
The dynamics of histone H2B-GFP during inhibition of rRNA synthesis in living HeLa cells. This gallery of optical sections was extracted from z-stacks of corresponding time series and displayed with 15–45 min intervals to fix the main stages of ICC evolution (corresponds to S8 Movie). The nuclei of three cells in the white rectangle on the initial image (“Start of experiment”) were enlarged to visualize the evolution of the ICC in more detail. In the course of AMD treatment we observed a gradual condensation of filamentous structures of the ICC into coarse clumps during 120 min with their migration from the nucleolar interior towards the PCC shell (165 min). During this movement several ICC clumps approached each other and fused just before the coalescence with the PCC. Note that at the end of the experiment (180 min) there was no more chromatin within the nucleolus. The scale bars represent 12 μm.

To further understand the precise reorganization of nucleolar chromatin, we performed time-lapse imaging of living HeLa cells expressing histone H2B-GFP. The displacement of ICC inside the nucleolus during AMD treatment is shown in movies (S7 and S8 Movies) and a gallery displaying 2D images of the corresponding movie (Fig 6). Initially (start of experiment, Fig 6A) all nucleoli revealed fluorescence mostly dispersed in the form of a fine meshwork, consisting of intermittent thin filaments and small clumps that always contacted the PCC. Within the first 15 min drastic changes in the localization and morphology of chromatin were observed; the filamentous structure began to condense into coarse aggregates (15 min, Fig 6B) and we observed a progressive condensation and increase of thickness of the PCC shell. After 30 min, we observed a significant disorganization of the intranucleolar meshwork: the filamentous appearance of histone H2B-GFP labeling was replaced by large clumps (30 min and 45 min, Fig 6C and 6D). Simultaneously these large clumps shifted from the nucleolar interior towards the PCC. During this migration several clumps approached each other and fused before they coalesced with the PCC shell (45 min, 60 min and 90 min). Thus, the number of GFP labeled intranucleolar structures decreased gradually. Next, the ICC shifted completely to the periphery and appeared like PCC protrusions into the nucleolar body (105–180 min). Frequently, fluorescence of H2B-GFP was no longer visible in the nucleoli.

### The intra- and perinucleolar condensed chromatin network is always in contact with structures containing UBF during nucleolar reorganization

**Fig 7:**
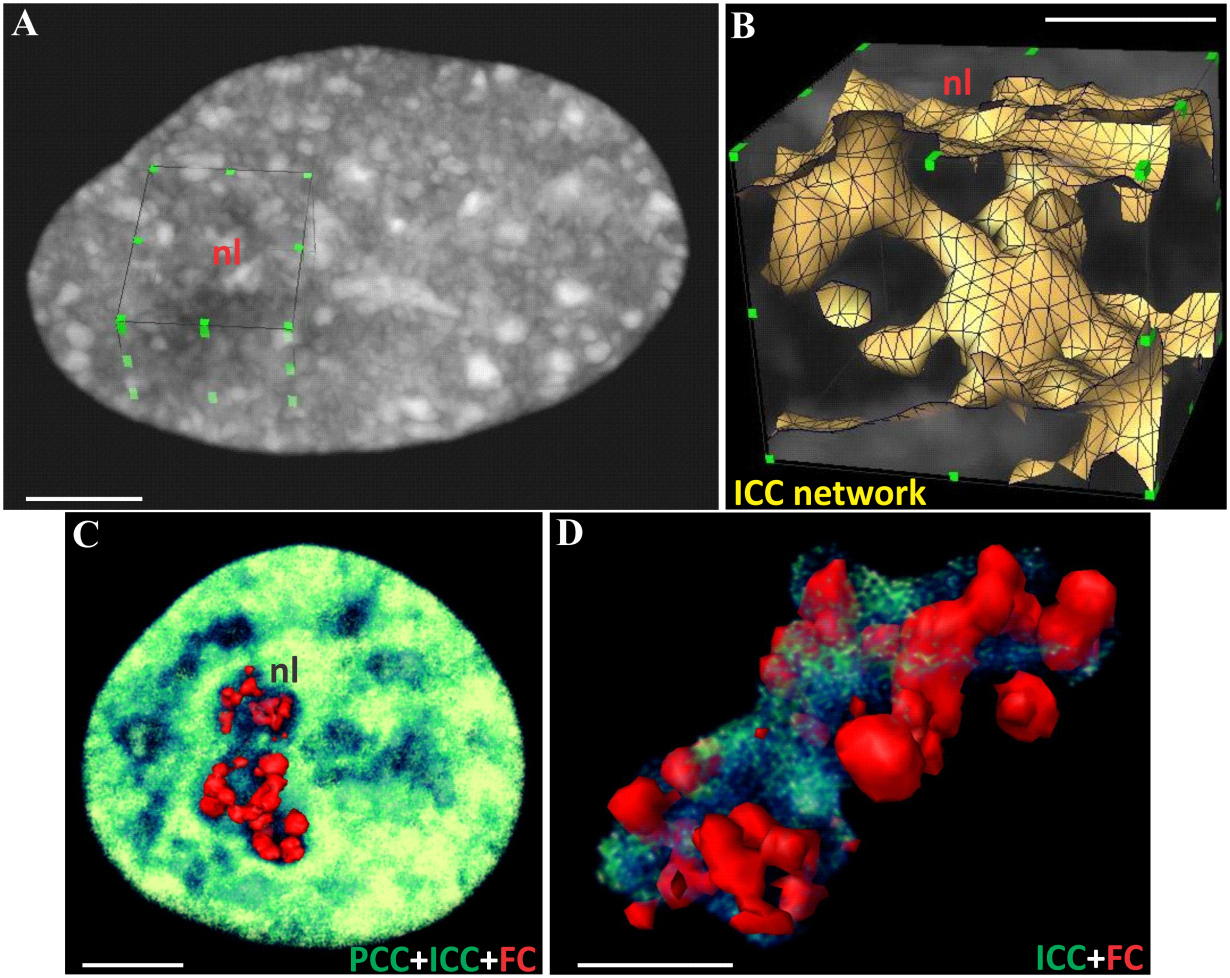
Spatial proximity of intranucleolar chromatin (ICC), perinucleolar chromatin (PCC), and UBF spots in fixed control cells. (A) 3D reconstruction of the nucleus in a HeLa cell stably expressing histone H2B-GFP using volume rendering. A virtual cube inserted into the nuclear volume delineates the nucleolar territory (nl) extracted in order to analyze its interior. (B) The nucleolar volume reconstructed using simultaneous volume rendering and surface visualization of intranucleolar H2B-GFP for better visualization of ICC. After volume extraction, the nucleolar interior revealed a network of ICC interconnected with the PCC shell. (C, D) Relationships between ICC (green) and UBF spots (red) within the nucleolus of KB cells cotransfected with H2B-GFP and UBF-dsRed. These 3D views demonstrate, inside the nucleolus, the presence of the ICC network in contact with UBF spots. The scale bar represents 3.5 μm in (A, B); 5 μm in (C) and 3.5 μm in (D).

To analyze the 3D relationships between the nucleolar condensed chromatin and structures containing UBF (termed UBF spots hereafter) we imaged immunolabeled UBF and H2B-GFP (or UBFdsRed and H2B-GFP) in fixed cells. In 3D views of control cells (Fig 7; S5 Fig), ICC appeared as an intra-nucleolar network connecting UBF spots with the PCC shell. This clearly demonstrated the proximity of ICC and UBF spots and the spatial integration of the latter into the whole network of NAC.

**Fig 8:**
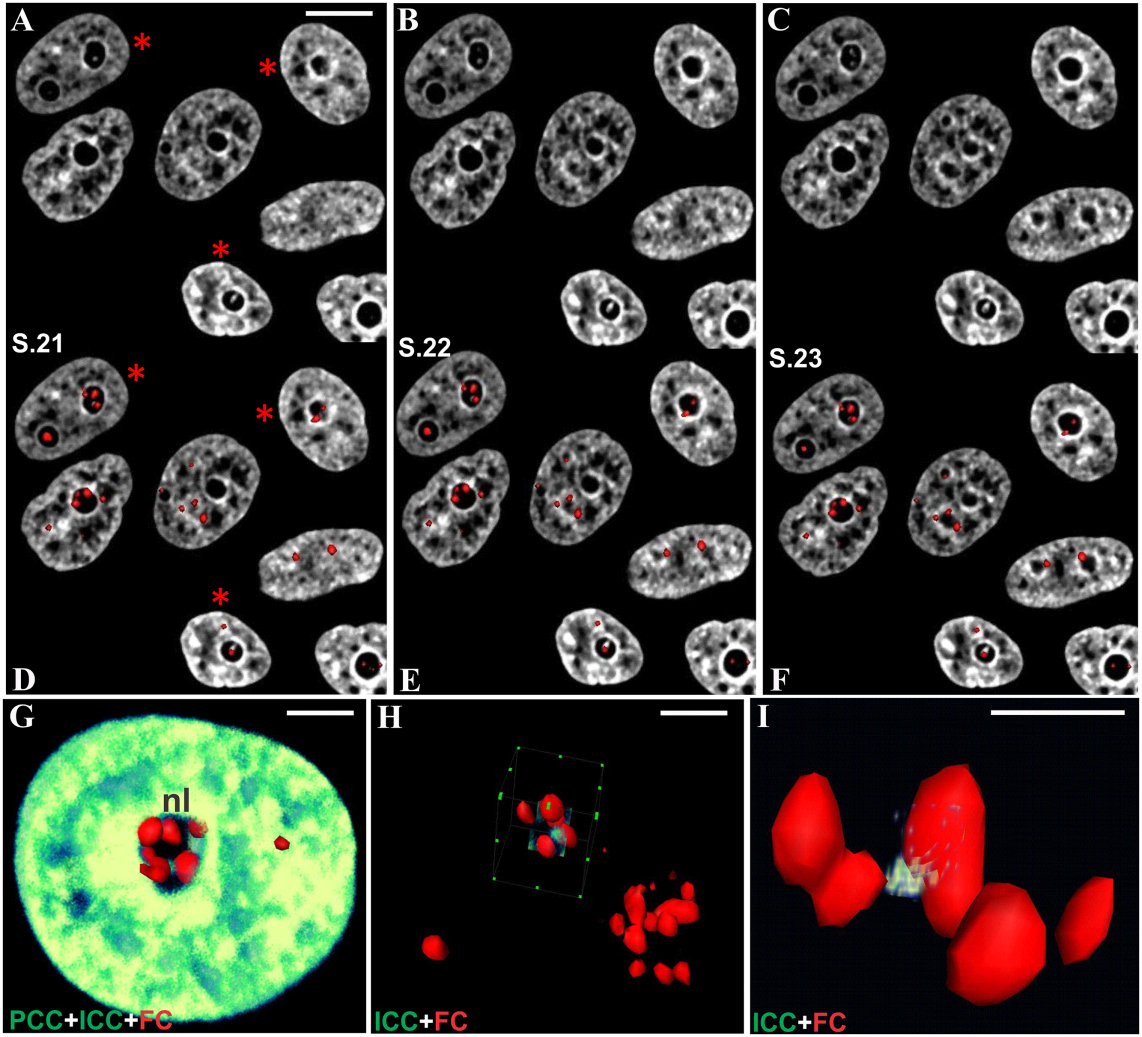
Interaction between UBF spots, ICC, and PCC within the pre-segregated nucleoli of HeLa and KB cells. (A-C) Consecutive optical sections (S.21-23) of nucleoli in HeLa cells stably expressing histone H2B-GFP. Note the prominent ICC clumps within the nucleolar interior after rRNA synthesis inhibition. These cells were immunolabeled for UBF at the end of time-lapse imaging (the corresponding nuclei are marked by red stars on A, D). (D-F) The same optical sections merged with 3D surface rendering of UBF spots (red). Note the direct contact between UBF spots and ICC inclusions. At the same time UBF spots that shifted to the nucleolar periphery became incorporated into the solid PCC shell. (G-I) 3D reconstructions of enlarged UBF spots extracted from the nucleolus of a KB cell doubly transfected with H2B-GFP and UBF-dsRed plasmids, fixed and imaged. A typical pre-segregated nucleolus containing five large UBF spots reconstructed using volume rendering (green) and surface visualization (red). Incorporation of peripherally-located UBF spots into the massive PCC shell as well as their contact with ICC can be readily recognized. Note the ICC clump that is in contact with two UBF spots. After extraction according to the virtual cubes in H, images were rotated at an appropriate angle to exemplify how UBF spots can be linked to each other by ICC. The scale bars represent 10 μm in (A); 5 μm in (G, H); 2 μm in (I).

This spatial interaction between UBF spots and histone H2B-GFP could be also demonstrated on 3D reconstructions of nucleoli of cells treated with AMD during 1 h (Fig 8). Moreover we also observed that ICC clumps, which were linked to UBF spots, were also in contact with a PCC shell and created a bridge between all these components. On the other hand, the same analysis performed on fully segregated nucleoli (2 h of AMD treatment) confirmed that there was no more ICC and that the convex side of the large UBF-positive caps was in contact with PCC.

Altogether, this analysis of fixed cells shows the permanent contact between UBF spots and ICC clumps during nucleolar reorganization.

### Ultrastructural imaging shows that UBF is always contained within FCs during inhibition of rRNA synthesis

**Fig 9:**
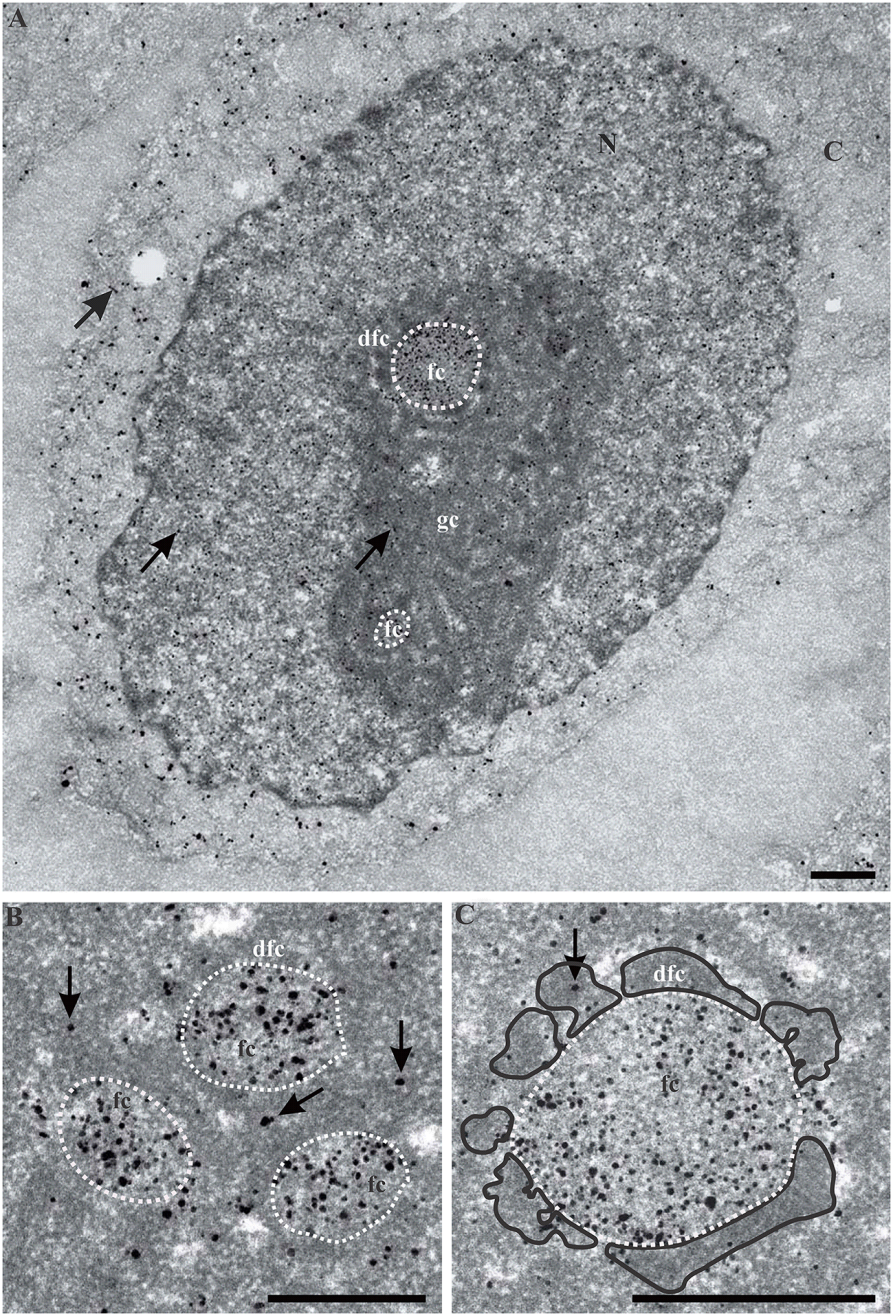
Ultrastructural pre-embedding localization of UBF within the nucleolus of control KB cells. (A) An abundant labeling (silver/gold particles) inside several fibrillar centers (fc) (outlined by a white dotted line). A few particles (black arrows) were visible within the nucleolar dense fibrillary component (dfc), nucleolar granular component (gc), nucleoplasm, or cytoplasm. (B, C) At high magnification, numerous particles were located within fibrillar centers (fc) (outlined by white dotted lines). The dfc is outlined by a black line on C. The scale bars represent 500 nm.

To identify the nucleolar structures which contain UBF during the reorganization induced by AMD, we immunolocalized UBF-GFP molecules and imaged them by electron microscopy, using a pre-embedding technique [35]. In control cells (Fig 9), numerous silver/gold particles identifying UBF-GFP were localized in FCs (limited by a dotted line) lined by a discontinuous cord of DFC (delimited by a black line on Fig 9C). Very few particles were identified within the DFC, granular component (GC), nucleoplasm, or cytoplasm.

**Fig 10:**
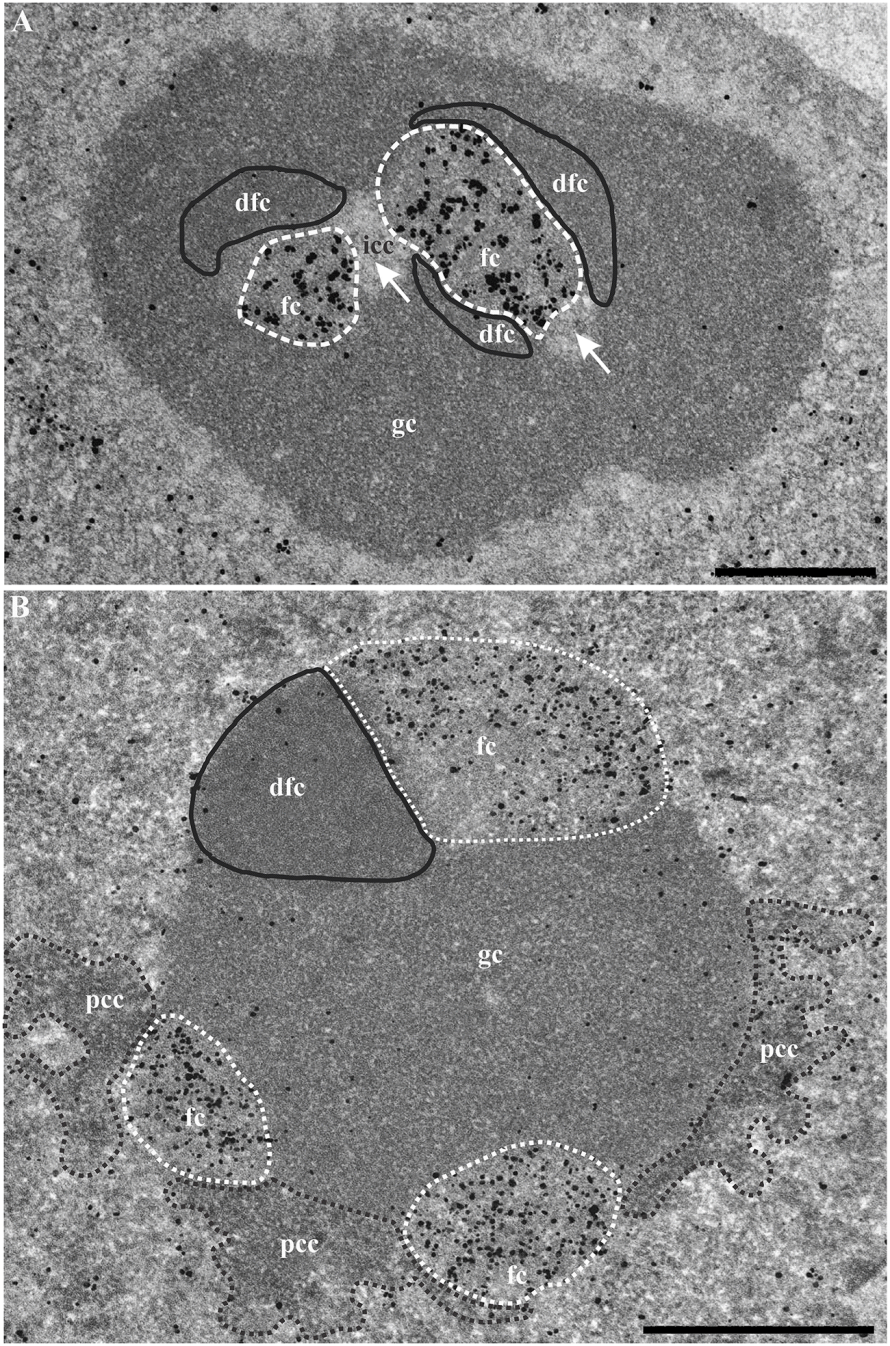
Ultrastructural pre-embedding localization of UBF within the nucleolus of KB cells treated with AMD for 1 h (A) or 2 h (B). (A) Abundant labeling (silver/gold particles) inside two large ovoid fcs (outlined by a white dotted line). White arrows point to electron-transparent vacuoles (or interstices) containing small clumps of intranucleolar condensed chromatin (icc). The dfc is outlined by a black line. (B) Numerous silver/gold particles were located within three cap-shaped fcs (outlined by white dotted lines) positioned at the nucleolar periphery. Note the direct contact between large clumps of perinucleolar condensed chromatin (pcc; outlined by a dark dotted line) and two fcs. Very few particles were found on the dfc (outlined by a black line) and the gc. Scale bars represent 500 nm.

After 1 h of treatment with AMD (Fig 10A), silver/gold particles identifying UBF-GFP were exclusively localized within FCs which were positioned close to each other and were frequently linked to vacuoles or interstices (arrows) containing small clumps of ICC. Contrary to control cells, the DFC partly surrounded the FC and was thicker; moreover the GC was more homogeneous and compact. After 2 h (Fig 10B), UBF-GFP labeling was found in cap-shaped FCs located on the periphery of ovoid and compact nucleoli. A direct contact between these FCs and large clumps of PCC was frequently observed.

### Successive time-lapse confocal microscopy and ultrastructural imaging of the same cells shows that condensed intranucleolar chromatin and fibrillar centers are close together and are contained within nucleolar vacuoles

**Fig 11:**
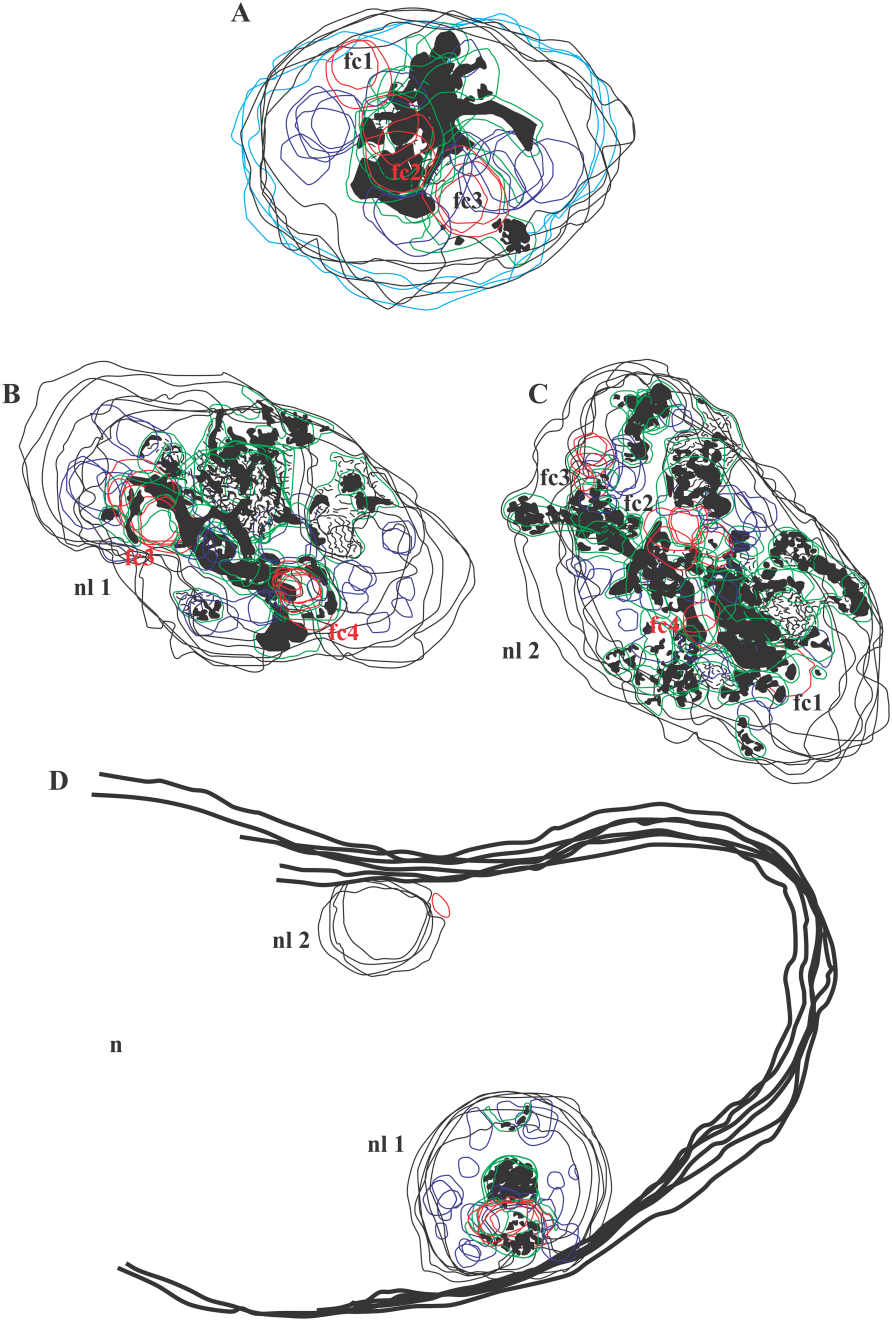
CLEM approach: Step 4. **Within cells treated with AMD during 1 h, the contours of nucleolar components identified on serial ultrathin sections were superposed to build 3D models of four different nucleoli (nl)**. Confocal images of the same nucleoli were used to confirm the identification of ICC (GFP fluorescence) and of FC (UBF fluorescence). The color-code is: nuclear and nucleolar contours—black and light blue lines; FCs—red and brown lines; ICC—black shading; NVs—green lines; DFC—dark blue lines. (A) 3D reconstruction of the equatorial part of the nucleolus in cell N°1(serial ultrathin sections on S10 Fig and serial fluorescent images shown on S14D and S14E Fig). (B, C) Two pre-segregated nucleoli in cell N°2 (serial ultrathin sections on S11 and S12 Figs and serial fluorescent images shown on S14A–S14C Fig). (D) 3D reconstruction of a nucleus with a ring-shaped nucleolus with one FC (serial ultrathin sections on S13 Fig and serial fluorescent images shown on S14F–S14I Fig).

To analyze the ultrastructure of nucleolar components in which ICC clumps observed in living cells are localized during the pre-segregated stages of AMD action, we developed a correlative light and electron microscopy (CLEM) approach (Fig 11, S6 and S17 Figs). The different steps of this approach were:

1. step 1 (S6 and S7 Figs): a group of COI (red circle on S6A–S6F and S7 Figs) was imaged by time-lapse confocal microscopy to analyze the AMD-induced 3D reorganization of intra-nucleolar chromatin tagged with H2B-GFP;
2. step 2 (S8 Fig, S9 Movie): after 1 h of AMD treatment, cells were fixed and immunostained for UBF. The same COI were then imaged by confocal microscopy to perform a simultaneous 3D visualization of ICC and UBF;
3. step 3 (S9 Fig): the cells were processed for electron microscopy and the COI were identified and trimmed; these cells were then cut through numerous 0.1 μm serial ultrathin sections and their nuclei were imaged at high magnification (S10–S13 Figs);
4. step 4 (Fig 11, S14–S17 Figs): the contours of nucleolar components identified on the serial ultrathin sections were superposed to build 3D models of different nucleoli treated with AMD during 1 h. Confocal images of the same nucleoli were used to confirm the identification of ICC (GFP fluorescence) and of FCs (UBF fluorescence)

Altogether, this overlay technique (CTO) allowed the demonstration that ICC clumps and FC are always close together during all the steps of nucleolar reorganization and are contained within a complex network of nucleolar vacuolar channels.

## Discussion

There is substantial evidence attesting that the nucleolus is largely involved in some specific events far beyond its key function as a pre-ribosome factory; it appears to house and integrate many versatile functions, playing a central role in the control of gene expression during cell cycle progression, aging, apoptosis, cellular stress signaling and responses, malignant transformation, viral infection, etc. [9–11, 63–77]. Over the past 15–20 years the nucleolus has become also recognized as a unique model to study the spatial organization of actively transcribing mammalian genes in the functional and dynamic association with overall structure and compaction of interphase chromatin. The molecular organization of the nucleolus is well documented at the genome and proteome levels. Conversely, it is still unknown how a giant tandem array of r-gene sequences associated with r-chromatin protein machineries is folded three-dimensionally to be structurally and functionally integrated within interphase NOR-bearing chromosomal territories. It remains much more problematic to understand how the structure and proper territorial arrangement of r-chromatin domains reorganize upon transcriptional inactivation of r-genes and how this is reflected in the dynamics of nucleolar components. Meanwhile, it has become generally accepted that the sophisticated spatial system of naDNA comprises transcriptionally active rDNA loops packed as non-nucleosomal structures and folded into FCs, along with nucleolar nucleosomal chromatin with still undetermined structure (genome map), functional activity and territorial organization. Once constituted, the nucleolus remains intimately associated with the physiological state of NAC, whose structural remodeling can affect the spatial arrangement of active r-genes and the global organization of the nucleolar factories.

However, the response of the whole naDNA system to the selective transcriptional arrest of r-genes in living cells remains completely unexplored. Therefore, the primary goal of this study was to demonstrate the structural and functional interplay between r-gene inactivation and large-scale modifications of intranuclear and intranucleolar structure including: (i) possible territorial reorganization of rDNA genes; (ii) nucleolar movement and intranucleolar dynamics of r-chromatin within FCs, and (iii) the role of NAC in the mechanism of intranucleolar reformation during segregation and capping.

### Are nucleoli “immobile” due to the fixed position of rDNA genes within NOR-bearing chromosomal territories?

It is known that active r-DNA genes occupy and share, together with mRNA genes, a central position in the nucleus [19, 78]. In the present study we showed that during segregation and capping, nucleoli do not change their initial relative positions within the nuclear volume, so that nucleolar relocation and fusion was never observed. In addition, FC and DFC from different nucleoli never gathered or fused, always staying within the limits of their own nucleolar territory. Consequently, each nucleolus may represent a separate compartment emerging due to the spatial interplay between r-chromatin loop(s) and nucleolar associated chromatin belonging to distinct NOR-bearing chromosomal territories that include ribosomal and non-ribosomal genomic regions. These findings imply that active and inactive nucleoli have a similar central location within the nucleus. This could result from the attachment of corresponding NOR-bearing chromosomal territories to the nuclear matrix that plays a key role in the compartmentalization and therefore in the function of the nucleus [79].

### Concerted contraction of nucleolar associated chromatin puts nucleolar components in motion

The ability of FCs and DFCs to move and fuse within a crowded nucleolar volume was revealed by early EM studies on artificial inactivation of r-genes, including the first experiments where AMD was used as an inhibitor [1–3, 52–54]. However, the mechanisms underlying spatial displacement of r-genes and transcription sites, as well as the sites of pre-rRNA processing at molecular and structural levels, have not been elucidated so far. Our interpretation of the dynamics of intra-nucleolar inactivation involves the mobility of the whole nucleolar-associated chromatin system on the one hand, and its structural integrity with the FC and adjacent DFC (hereafter FC/DFC assembly) on the other. It is the simultaneous contraction of the ICC and PCC and perinucleolar dislocation of ICC that induce the motion of the FC/DFC assembly ending in the form of cap-shaped structures tightly adherent to the PCC rim.

Contrary to a relatively fixed position of rDNA genes in response to their selective inhibition, we observed clear intradomain dynamics. Therefore, together with strong rotational movement of the nucleolus we traced the intranucleolar dynamics of FCs, particularly, their initial (within 1 h) confluence into larger structures, and followed up their further migration from the center of the nucleolus to its periphery. The motion of individual FCs was apparently asynchronous.

As a rule, large FCs localized on the periphery of the nucleolus remained immobile for a long time until their final transformation into caps. At the same time, smaller FCs, more dispersed throughout the nucleolar volume, were continuously migrating. The structures resulting from the fusion of single small FCs gradually moved up to approach the larger “immobile” FCs, first coming closer and then merging with them.

ICC clumps also demonstrated similar trajectories and asynchrony, whereas large peripherally located ICC blocks bound to the PCC rim exhibited low mobility. Analysis of the movies shows that the disorganization of the intra-nucleolar chromatin network involved the gradual gathering of the filamentous structure of ICC into coarse aggregates and their shift from the nucleolar interior towards the PCC. Like FCs, in later steps of AMD action, ICC clumps gradually shifted to a perinucleolar location. However, peripherally located ICC clumps, before they completely merged with the PCC rim, were capable of keeping a long-lasting contact with the PCC and although relatively “immobile”, they still remained clearly visible as distinct intranucleolar structures. At the same time, new blocks from the inner regions of the nucleolus started to move closer to them because of further condensation of the unit ICC network. They either merged with larger ICC clumps or, having reached the nucleolar periphery, stayed “immobile” and visible within a certain period of time until incorporated into PCC. These live cell observations strongly support our proposition that gradual coalescence events are largely involved in the mechanisms of nucleolar segregation and capping.

### Is intranucleolar dynamics of FCs restricted due to attachment to PCC via DJ heterochromatin?

The dynamics of FCs observed during time-lapse imaging can be explained by the progressive condensation of nucleolar-associated chromatin in response to inactivation of r-gene transcription. The physical bond between FCs and ICC may provoke their movement due to the dynamics of nucleolar-associated chromatin constituents. Thus within incompletely segregated nucleoli, FCs were always in contact with intranucleolar blocks of chromatin, which in turn were connected with PCC that forms a thick layer around an inactivated nucleolus. The position of peripherally shifted ICC blocks is reminiscent of that of flanking rDNA heterochromatin protrusions, DJ in particular [25]. This suggests that ICC may represent protrusions of PJ and DJ into nucleolar territory whereas PCC blocks surrounding the nucleolar territory may be constituted by centromeric and telomeric heterochromatin. Finally, the disappearance of ICC (observed on Figs 5 and 6) could result from its incorporation into PCC. After this step, FCs spread onto the surface of the residual nucleolus and we propose that they acquired a crescent-like shape due to their squeezing between the PCC rim and the spherical body of the residual nucleolus.

### Dynamic model of the nucleolus

Now, our aim is to formulate a dynamic model embracing the data about a possible structural and functional impact of nucleolar-associated chromatin and of the vacuolar component in the global organization and inactivation dynamics of the nucleolus. By merging fluorescence and EM images we reached the conclusion that r-chromatin of FCs, together with intra- and perinucleolar condensed chromatin, is part of a unit system structurally integrated into the network of nucleolar vacuoles. Importantly, during the selective inactivation of r-gene transcription this system reacts in a concerted manner, so that 3D reorganization of the vacuolar system proceeds in parallel with the rearrangement of the FC/NAC unit. Therefore we consider nucleolar vacuoles rather as an active nucleolar constituent, crucial for the spatial distribution of ICC and FCs. In this context we hypothesize that continuous ICC strands, emanating from the PCC shell, are interconnected with FCs through the network of united channels attributed on ultrathin sections to the vacuolar component.

The unit system described comprises the UBF-positive non-nucleosomal segments of r-chromatin, loosely folded at the transcription sites, as well as nucleosomal chromatin located between FCs and at the border with PCC. In contrast to nucleosomal chromatin, the relaxed state could be considered as ubiquitous, facilitating transcription and replication within FC. Meanwhile, nucleosomal intra- and peri-nucleolar condensed chromatin regions are physically connected with r-chromatin and may contain silenced r-genes as well as non-ribosomal genes. At least the portion of intra-nucleolar chromatin seems to be constructed from centromeric and telomeric heterochromatin segments linking respectively to the proximal and the distal rDNA flanking regions.

Thus, the functional organization of r-chromatin within the nucleolar space can exist due to the spatial interplay between rDNA and heterochromatic blocks emanating from peri-centromeric and peri-telomeric segments of the short chromosome arms. We can also propose that during nucleologenesis [80] the centromeric and telomeric heterochromatin blocks of several juxtaposed NOR-bearing chromosomal territories can interact, forming a PCC shell separating the nucleolar volume from the nucleoplasm. Then, it could be well assumed that the permanent repressive and heterochromatinized state of ICC or of the entire NAC may appear to provide the force ensuring the global integrity of the nucleolus.

Furthermore, peripherally-shifted ICC clumps directly linked to the surrounding PCC can be attributed to the peri-telomeric DJ regions, while ICC localized inside the nucleolar volume corresponds to peri-centromeric PJ chromatin. This sophisticated structure provides the structural support for spatial organization of the nucleolus.

The obvious implication of this model is the possibility of schematizing the pathway of nucleolar reorganization during inhibition of rRNA synthesis. During this inhibition the size of the nucleolus gradually decreases. Moreover, we propose that the density of compaction within intra-nucleolar chromatin increases with the inactivation of r-genes. Thus we can hypothesize that the chromatin of DJ origin positioned in the peripheral sites of intra-nucleolar condensed chromatin meshwork becomes more condensed, then shortened and shifted to PCC due to its contraction. During their migration towards PCC they also pull the connected FCs to the nucleolar periphery. Peri-centromeric intra-nucleolar condensed chromatin from the nucleolar interior, localized between FCs, becomes more condensed as well. As a result, the distance between FCs also decreases, so that FCs may juxtapose within the pre-segregated nucleoli. The juxtaposed FCs can be readily recognized as individual entities because they are still separated by ICC clumps. Very likely the physical/chemical state and therefore the dynamics of individual FC/DFC assemblies, enriched by UBF and pre-rRNA, can be similar to those of self-assembling soft active aggregates that are characterized by a high concentration of proteins and RNAs [81]. Hence, by using a targeted cryo-correlative nano-imaging approach, we recently demonstrated that DFC and FC of control cells contained around 70% and 80% of water, respectively [82]. In the same study, we also quantified the water content in cells treated with AMD for 3 h and found an increase of water content in all compartments of the nucleus and cytoplasm and, more particularly, that segregated DFC and FC (caps) contained around 80 and 90% of water, respectively. Thus, we propose that the increase of water content induced by AMD treatment could decrease the viscosity of nuclear compartments and facilitate the reorganization and fusion of chromatin clumps, of DFC and FCs as described for different types of protein- and RNA-rich NBs considered as condensed liquid-like droplets [29, 81]. Finally, ICC clumps inserted between FCs fused with the PCC shell, provoking coalescence of individual FCs and their gathering as nucleolar caps on the nucleolar periphery. This suggests that each nucleolar cap might be composed of several FCs juxtaposed on the margin of the nucleolar remnants.

### Concluding remarks

In the present study we used several complementary cell imaging approaches to provide new insights into the spatial reorganization of r-genes upon AMD inactivation, relative to condensed chromatin with a canonical structure. Time-lapse confocal microscopy showed that: i) nucleoli remain independent units upon inhibition of rRNA synthesis, ii) in each nucleolus, structures containing UBF gather during a sequential process and are always in contact with the condensing network of intra and peri-nucleolar chromatin. Successive time-lapse confocal microscopy and ultrastructural imaging of the same cells demonstrate that all FCs contain UBF and are always in contact with condensing intra-nucleolar chromatin. Finally, the finding that FCs and intra-nucleolar chromatin are contained within large nucleolar vacuoles suggest that they are in continuity and belong to the same chromatin entity.

## Supporting information

S1 Fig Structural and ultrastructural features of nucleoli in control KB and HeLa cells. (A) KB cells imaged by phase-contrast microscopy. Note the prominent light zones (outlined by white circles) within the large dense nucleoli (nl). (B) In EM images, nucleoli of KB cells exhibit a clear tripartite organization that includes: (i) large, pale-stained fibrillary centers (FC); (ii) a well-developed rim of dense fibrillary component (DFC) tightly encircling FCs (FC/DFC assembly); and (iii) an abundant granular component (GC) of moderate density. Numerous electron lucent interstices (arrows) containing intra-nucleolar condensed chromatin (ICC) fibrils and clumps penetrate the GC. All nucleoli were found in association with large blocks of perinucleolar condensed chromatin (PCC). (C) Confocal microscopy imaging of nuclei in HeLa cells stably expressing histone H2B-GFP. Note the profound rim of PCC (arrows) surrounding the nucleolar territory (nl). Nucleoli contain numerous ICC inclusions that link to the PCC shell. The scale bars represent 4.5 μm in (A); 1.5 μm in (B); 10 μm in (C).
(TIF)

S2 Fig Presence of the ICC network inside nucleolar volume of KB cells. (A, B) X/Y and Z/Y optical sections of the nucleus in a cell transiently expressing H2B-GFP. C) X/Y section of the nucleus in the same cell as (A) showing the nucleolus (nl) at higher magnification. Arrows indicate strands of ICC. (D, E) Two consecutive Z/Y sections of the nucleus shown on the preceeding images. Arrows point to ICC strands localized in the depth of the nucleolar volume. The scale bars represent 5 μm in (A, B); 3 μm in (C); 3.5 μm in (D, E).
(TIF)

S3 Fig 4D evolution of nucleolar volume in the course of AmD treatment. Corresponds to the cell presented on S1–S5 Movies. (A—H) Gallery of 3D reconstructions displaying nucleolar changes during the inhibition of rRNA synthesis. These reconstructions were performed using surface rendering at medium threshold to show nucleolar limits. Nucleoli, with an initial irregular shape, became spherical during inhibition. The scale bar represents 2 μm.
(TIF)

S4 Fig 2D/3D organization of UBF-GFP in control KB cells (CTRL) and cells treated with AMD for 1 h (AMD 1H) or 2 h (AMD 2H). (A, D, G) phase contrast. (B, E, H) merged phase contrast and fluorescence. (C, F, I) 3D reconstruction of UBF-GFP fluorescence. In control cells, UBF was localized exclusively in the form of brightly fluorescent spots juxtaposed in a chain-like manner. In cells treated with AMD, UBF was localized strictly within large nucleolar regions with a low phase contrast (arrows on D and G). The scale bars represent 5 μm.
(TIF)

S5 Fig Continuity of UBF-positive structures and of nucleolar associated chromatin (NAC) in fixed control HeLa cells. (A) 3D reconstruction of the nucleus (green) and immunolabeled UBF (red) in cells stably expressing H2B-GFP (transparent surface rendering). Black circles delineate the nucleoli (nl). (B, C) Two successive virtual sections (X/Y planes) revealing a strong PCC shell surrounding the nucleolus. Profound ICC strands (blue arrow) which are in a close structural link with UBF-positive NCs (S3B Fig) look like protrusions of PCC into the nucleolar space (S3C Fig). (D–L) Gallery of successive virtual sections cut in X/Y (S.28-32; S3D–S3H Fig) and X/Z (S.253-256; S3I–S3L Fig) planes shows the incorporation of ICC clumps with UBF-positive NCs on one side and ICC with PCC on another side. The close structural link between ICC (blue arrows) and UBF-positive NCs is obvious when imaged at different depths of cutting (yellow arrows). The scale bars represent 5 μm.
(TIF)

S6 Fig Correlative Light and Electron Microscopy (CLEM) approach: Step 1. (A–F) Start of experiment HeLa/H2B-GFP cells imaged before addition of AMD. Cells selected for CLEM are localized in red circles. (G, H) At higher magnification, the nucleoli (nl) were seen distinctly by Nomarsky contrast; fluorescence imaging of H2B-GFP revealed intranucleolar clumps of ICC. Four cells were identified (#1 to #4). The scale bars represent 100 μm in (A-C); 50 μm in (D-F); 10 μm in (G, H).
(TIF)

S7 Fig Correlative Light and Electron Microscopy (CLEM) approach: Step 1 (continued from S6 Fig). (A–F) End of experiment: the same ROI as on Fig 6A–6H imaged after treatment with AmD during 1 h. The topography of cells selected for CLEM (red circles) remains unchanged. (G, H) At higher magnification, the nucleoli (nl) were seen distinctly by Nomarsky contrast; fluorescence imaging of H2B-GFP revealed coarse intranucleolar clumps of ICC. Four cells (N°1 to N°4) were selected for CLEM. The scale bars represent 100 μm in (A-C); 50 μm in (D-F); 10 μm in (G, H).
(TIF)

S8 Fig CLEM approach: Step 2 (continued from S6 Fig). (A-H) The COI on S6 Fig was fixed after 1 h of AMD treatment (S8A–S8G Fig), immunolabeled for UBF (S8H Fig), and imaged by confocal microscopy. The localization of cells within the target group was the same as during living cell imaging. (G, H) At higher magnification after fixation the nucleoli, imaged in Nomarsky contrast, became prominent (G). All pre-segregated nucleoli (nl) reveal several UBF-positive red label (H). All the nucleoli revealed UBF-positive red label (H). The same four cells (N°1 to N°4) were selected for CLEM. The scale bars represent 100 μm in (A-C); 50 μm in (D-F); 10 μm in (G, H).
(TIF)

S9 Fig CLEM approach: Step 3 (continued from S6 Fig). (A, B) After dehydration and plastic embedding, serial ultrathin sections of the same group of cells were imaged by electron microscopy. (A) H2B-GFP is green and UBF is red. All the nucleoli revealed UBF-positive red label. Note the structural continuity of ICC and UBF-positive spots. (B) On this section cells 1 to 4 are clearly identified (compare with S9A Fig). Nucleolar sub-components of cells N°1 and N°2 (marked by yellow stars) are shown in more detail in S10–S12 Figs. The scale bars represent 12 μm in (A); 16 μm in (B).
(TIF)

S10 Fig CLEM approach: Step 3 (continued from S6 Fig). The pre-segregated nucleolus of the cell N°1 (yellow star on S9 Fig) was analyzed by electron microscopy through 21 serial 0.1 μm ultrathin sections. (A-J) Ten of these sections are shown. This ultrastructural analysis clearly identified four FCs (FC N°1 to N°4; outlined by dotted black lines) which appeared as circular structures surrounded by NVs and only partly in contact with reorganized dense fibrillary component (dfc). Note that clumps of intranucleolar condensed chromatin (icc) were observed within vacuoles. Granular component (gc); peri-nucleolar condensed chromatin (pcc). The scale bars represent 0.5 μm.
(TIF)

S11 Fig Seven serial ultrathin sections (of a total of 17) of one of the two nucleoli in the cell N°2 submitted to CLEM analysis. (A–G) The nucleolus localized in the left part of the nucleus (see S9 and S14A–S14C Figs) contains 5 FCs, but only 3 appear within the serial sections shown. Note that two FCs (FC N°3 and FC N°4) are connected by an ICC strand localized within the narrow channel belonging to the NV (D). The scale bar represents 0.5 μm.
(TIF)

S12 Fig Seven serial ultrathin sections (a total of 17) of one of the two nucleoli in the cell N°2 submitted to CLEM analysis (continued). (A–G) The nucleolus localized in the right part of the nucleus (see S9 and S14A–S14C Figs) contains 4 FCs. The nucleolus is entirely penetrated by the system of expanded NV in which FCs and ICC are localized. The structural link between FCs and intravacuolar ICC inclusions is obvious. The scale bar represents 0.5 μm.
(TIF)

S13 Fig Six serial ultrathin sections (of a total of 28) of a nucleus containing a ring-shaped nucleolus. (A-F) Successive ultrathin sections (S.10–S.15) crossing the “equatorial” segment of the nucleolus that shows a so-called ring-shaped organization of NCs. Corresponds to the cell presented on S14F–S14I Fig (matched by red star). The large FC was separated by a black dotted line on (B-F). Note that the FC is localized within a large, round NV filled with ICC. Thus the unity of FC, ICC and NV seems to be a common feature for all nucleolar types. The scale bar represents 1.5 μm.
(TIF)

S14 Fig CLEM study: Spatial relationship between FCs and ICC in pre-segregated nucleoli. These images represent HeLa cells treated with AMD during 1 h. To correlate FCs and ICC with the structures fluorescently marked by anti-UBF antibody and H2B-GFP, we applied the confocal-transmission electron microscopy overlay technique (CTO) on the same cells (cells N°1 and N°2 in S9 Fig). To arrest the nucleolar transformation before segregation we fixed cells after 1 h or 1.5 h and performed anti-UBF immunolabeling. (A-C) Serial optical sections were combined with surface rendering for UBF-positive NCs (red) to follow the link between FCs and ICC in light and electron microscopy. (D, E) The nucleus of the cell N°1 selected for CLEM analysis at higher magnification. (F-I) Serial optical sections of the nucleus with a so-called ring-shaped nucleolus. Serial sections revealed one large UBF-positive FC in contact with prominent ICC clump. The scale bar represents: 12 μm in (A, C); 6 μm in (D, E); 8 μm in (F-I).
(TIF)

S15 Fig Identification of nucleolar components by superposition of corresponding structures/ultrastructures on fluorescence and EM images. To perform CLEM, fluorescence images of the cell N°1 (S9 and S14A–S14E Figs) were rotated 90° to be properly aligned with the corresponding TEM images (S10 Fig). 3D reconstructions were performed by accurate matching as described in Materials and methods. (A1–4) Demonstration of structural relationship between condensed chromatin labeled by H2B-GFP (black) and anti-UBF antibody (red) using a successive colocalization approach. (A1) H2B-GFP forms a massive clump crossing the nucleolar volume (corresponds to S14D and S14E Fig). (A2) Due to lower resolution, only two UBF-positive NCs were distinguished at the LM level while in EM the corresponding cell reveals four FCs. (A3, A4) The structural link visible on the merged contours of H2B- and UBF-positive NCs repeats the picture observed on S14D and S14E Fig. (B1–5) 3D reconstructions performed by a successive colocalization of the contours taken from serial ultrathin sections of a corresponding nucleolus. (B1, 2) Colocalization of ICC and NVs (green): the massive ICC clump appears completely immersed in the complex network of NVs. (B3, 4) Colocalization of FCs (brown) with the network of NVs: FCs are always in contact with NVs. (B5) Colocalization of ICC, FCs and NVs (green): the unity of these NCs within the nucleolar volume is clear. (C1–3) The nuclear and nucleolar contours taken from the fluorescence images are marked in black and the nucleolar contours from serial ultrathin sections are marked in light blue. (C1) Localization of the fluorescent label (black) within the unit system of NVs (green) proves the identity of intra-nucleolar H2B-positive inclusions with ICC. (C2, C3) Perfect colocalization of the anti-UBF fluorescent label (red) with three FCs (brown) and NVs (green) definitively attributes UBF-positive structures to FCs. The 3D reconstruction in (D1) was constructed using the sections cut at the same depth along the z-axis as (A4). In both cases the massive ICC clump appears to be inserted between two FCs.
(TIF)

S16 Fig Identification of NCs by superposition of the corresponding structures/ ultrastructures on fluorescence and EM images (continued). 3D reconstructions according to the nuclear and nucleolar contours outlined on fluorescence and EM images of two nucleoli (nl N°1 and nl N°2) inside the cell N°2 (corresponds to the S14A–S14C Fig). (A1, 2) Visualization of the structural link on the merged contours of the nucleus and NCs fluorescently labeled by H2B-GFP (black) and UBF (red), using a successive colocalization approach. (B1–4) 3D reconstructions obtained by the successive colocalization of contours taken from serial ultrathin sections of the nucleoli N°1 and N°2 (corresponds to S9 and S14A–S14C Figs). Colocalization of FCs (red) with the network of NVs (green): FCs are largely in contact with the vacuolar component of the nucleolus. (C1–3) Colocalization of ICC (black) and NVs (green) performed for nucleolus N°1 (C1) as well as ICC, FCs and NVs in nucleolus N°2 (C2, C3) in order to demonstrate the unity of these NCs. (D1–4) CTO of fluorescent and TEM images adjusted to the same magnification. The nuclear and nucleolar contours taken from fluorescence images are marked in black and the nucleolar contours on ultrathin sections in light blue. Colocalization of fluorescent H2B-GFP label (black) within the unit system of NVs (green) proves the identity of intra-nucleolar histone H2B-positive inclusions and ICC (D1). The colocalization of fluorescent anti-UBF label (red) with FCs (brown) and NVs (green) indicates the identity of UBF-positive structures and FCs (D2, D3). At the same time the colocalization of the fluorescent histone H2B-GFP and anti-UBF labels with ICC, FCs and NV (D4) demonstrates the unity of these NCs.
(TIF)

S17 Fig Identification of NCs by superposition of corresponding structures/ultrastructures on fluorescence and EM images (continued). 3D reconstruction of the pre-segregated nucleolus in S13 and S14F–S14I Figs. Because of the presence of a large FC and a still profound NV these nucleolar modifications represent a suitable model to demonstrate the unity of the NCs. (A1–3) Successive colocalization approach to demonstrate the structural link between the massive intra-nucleolar block labeled by H2B-GFP (black) and UBF (red). (B1–3) EM colocalization of FCs (red), NV (green) and ICC (black): a massive ICC clump and FC are completely immersed in the NV (B3). (C1 –D3) merge of fluorescence and TEM images using a successive colocalization approach. The nuclear and nucleolar contours taken from the fluorescence images are marked by black, whereas identical contours outlined on the TEM images are light blue. Perfect colocalization of black and red fluorescence labels with ICC, FC and NV (green) proves the idea that these structures compose the unit system.
(TIF)

S1 Movie Dynamics of fibrillarin-GFP labeling: 8 h AMD treatment of living KB cells. This 2D movie was created using z-/time-series collected at 5 min intervals by a Bio-Rad MRC-1024E LCM and Amira 5.5 software. Corresponds to the cell in Fig 1 and S3 Fig.
(MOV)

S2 Movie 4D dynamics of nucleoli: 8 h AMD treatment of living KB cells. This movie was created using z-/time-series collected at 5 min intervals by a Bio-Rad MRC-1024E LCM and Rev4D software. Corresponds to the cell in Fig 1, S3 Fig and S1 Movie. During the experiment the shape of nucleoli changes from complex to spheroid but they retain their fixed position within the nuclear volume. No nucleolar fusion was observed.
(MOV)

S3 Movie 4D dynamics of nucleoli: 8 h AMD treatment of living KB cells. This movie was created using z-/time-series performed as in S2 Movie. Corresponds to the cell in Fig 1, S3 Fig and S1 Movie. The trajectory of the center of mass of each nucleolus is traced in space and time from left to right. During the last second of the movie, time is contracted in order to show the true 3D trajectory of each nucleolus within the nuclear volume. No nucleolar fusion was observed.
(MOV)

S4 Movie 4D dynamics of nucleoli: 8 h AMD treatment of living KB cells. This movie was created using z-/time-series performed as in S2 Movie. Corresponds to the cell in Fig 1, S3 Fig and S1 Movie. Trajectories of three nucleoli are traced in space and time from left to right. During the same time, the evolution of the volume of each nucleolus is traced showing a strong decrease beginning at time 1 h.
(MOV)

S5 Movie 4D dynamics of fibrillarin-GFP labeling within dense fibrillary component: 8 h AMD, treatment of living KB cells. This movie was created as S2 Movie. Corresponds to the cell in Fig 1, S3 Fig and S1 Movie.
(MOV)

S6 Movie Dynamics of UBF-GFP labeling: 8 h AMD treatment of living KB cells. This 2D movie was created using z-/time-series collected as in S1 Movie. Corresponds to the cell in Figs 2 and 3.
(MOV)

S7 Movie Dynamics of histone H2B-GFP labeling: 3 h AMD treatment of living HeLa cells. This 2D movie was created using z-/time-series collected at 5 min intervals by a Zeiss 710 NLO LSM and Zen 2011 and Quick Time software. Corresponds to the cells in Fig 6.
(MOV)

S8 Movie Dynamics of histone H2B-GFP labeling: 3 h AMD action on living HeLa cells (continued). This 2D movie was created using z-/time-series collected at 1 min intervals by a Spining Disk ILAS2 CS, Amira 5.5 and Quick Time software.
(MOV)

S9 Movie Correlative study: spatial relationship between FCs and ICC during nucleolar segregation. This 2D movie demonstrates the dynamics of intra-nucleolar histone H2B in the cells shown in S14D and S14E Fig. To show that during the entire process of segregation the ICC remains attached to FCs, observation was ended with fixation and anti-UBF/Alexa568 immunolabeling. To create this movie we used z-/time-series collected with a Zeiss 710 NLO LSM at 5 min intervals. Consecutive orthogonal slices of X/Y planes prepared for each acquisition point using Amira 5.5 were mounted by Quick Time software. After post-fixation immunolabeling the UBF-positive FCs were 3D reconstructed with Amira and visualized on the background of orthogonal sections of the histone H2B-GFP labeled nucleus. The corresponding image is shown at the end of the movie.
(MOV)

S1 Method Transient transfection/co-transfection. To prevent formation of aggregates the cell suspension was agitated in different directions and by circular motion during a few min. Cells were incubated for 24 h at 37°C to reach 50–60% confluence, examined on an inverted microscope to select samples, rinsed with PBS, and immersed in fresh medium for 2–4 h. The transfection complex containing 4.8 μg cDNA and 7.2 μl Fugene-6 was prepared in 200 μl serum-free medium, incubated 15 min at room temperature, and poured over the cells growing in the last change of medium.
(DOCX)

S2 Method Observation and imaging of fixed KB cells. Using a Bio-Rad MRC-1024ES/Olympus IX70 CM, GFP fluorescence was induced by excitation at 488 nm at 3–10% of Kr/Ar laser power in combination with VHS and Open filter blocks. Emission was recorded at 515 nm with an OG515 filter. In GFP/DsRed doubly-labelled preparations excitation was at 488 and 568 nm using 10% of laser power and T1 and T2 filter blocks, enabling simultaneous transmission of both bands and their recording through green and red channels (PMT2 and PMT1, respectively) with the following emission filters: for green 522DF35 (blocks 513 and 540 nm bands) and for red 605DF32 (blocks 589–621 nm). Images were acquired by Laser Sharp 3.2 software at zoom x4 corresponding to a pixel size of 0.080 μm, slow scanning speed mode (3 sec for each 512x512 image), and Kalman digital filtering (x3) to ameliorate signal/noise ratio. Phase contrast and fluorescence images were digitalized simultaneously. Consecutive optical sections (70–100 slices) were collected using 0.2 μm z-steps yielding stacks ~14–20 μm thick including entire KB cell nuclei. 3D localization of UBF and fibrillarin in fixed cells was reconstructed and visualized by Amira 5.5 software using two methods: (i) the “Isosurface” mode, displaying the exterior morphology (shape, number, and size) by surface rendering and (ii) the “Voltex” mode, representing interior organization by the rendering of the whole volume. 2D images were presented in “Maximum Intensity Projections” (MIP) mode after being processed using regular image-treatment and graphic software such as ImageJ, Corel Draw, and Photoshop.
(DOCX)

S3 Method Observations and imaging of living KB cells. Using a Bio-Rad MRC-1024ES/Olympus IX70 CM, series of 60 optical slices with a z-step of 0.35 μm were recorded at zoom x3 and Kalman x2 digital filtering to ameliorate the signal/noise ratio. Recording of one Z-stack took 75 sec at fast scanning speed, including time for displacement along the z-axis. To prevent bleaching and/or photo damage we reduced illumination by around 20%, exciting GFP by 3% power of the Kr/Ar laser with B1/Open filter blocks. Emission was recorded as for fixed cells. Time-/z-series were recorded every 5 mins for 8 h. After 30 min the cells were perfused with AMD which was replaced by fresh medium after 2 h and collection was continued for 5.5 h. To demonstrate the general features in the behavior of the components containing fibrillarin and UBF we used simple visualization tools in the form of 2D movies provided by ImageJ or QuickTime.
(DOCX)

S4 Method Preparation of KB cells for anti-GFP immunolabeling and silver enhancement. Cells were briefly rinsed with PBS, fixed for 10 min in 4% paraformaldehyde in PBS adjusted to pH 7.2–7.4, rinsed repeatedly in PBS 3x5 min, and processed for CM and pre-embedding anti-GFP-fluoronanogold immuno-EM. Cells were incubated in 0.5% Triton X-100 in PBS for 5 min, in 10% normal goat serum (NGS) (Jackson, USA) in PBS for 30 min, and then with mouse monoclonal anti-human GFP antibody (Roche Diagnostics) (1:50 in PBS) for 30 min. The cells were rinsed repeatedly (15 min) in the above solution of NGS and incubated with goat anti-mouse biotinylated secondary antibody (Jackson) (1:100) for 30 min. Secondary antibodies were detected by a 15 min exposure to streptavidin-fluoronanogold conjugate (1:20 in PBS). Labeled cells were then postfixed in 1.6% glutaraldehyde and washed 3x10 min in PBS to remove glutaraldehyde completely. Importantly, when silver enhancer was used all procedures were at room temperature in light-tight boxes. Before silver enhancement cells were washed extensively (10x2 min) in deionized water. Silver enhancement was carried out at room temperature (about 20°C) for 7–9 min; the quality of staining is strongly temperature/time-dependent. To eliminate background staining enhancement was arrested by rapid immersion in ice-cold deionized water. The cells were thoroughly washed again (10x2 min) in deionized water and treated for 10 min in 5% aqueous sodium thiosulphate to quench residual metallic silver. The quality of labeling was controlled by phase contrast microscopy at 40x10 magnification with, removing the RS40 diaphragm. In properly stained cells the UBF positive nucleolar sites are clearly recognizable as dark brown, folded bead-like chains or relatively large distinct spots in sharp contrast against the pale yellow color of the nucleoplasm.
(DOCX)

S5 Method Preparation of He-La cells stably expressing fibrillarin-GFP and histone H2B-GFP for time-lapse imaging. The concentration of cells must be adjusted to yield a culture sparse enough to facilitate localization of single cells or small groups. A satisfactory density on the finder grid was obtained if cells were seeded by the following 2 protocols (S6–S8 Figs). (i) The bottom glass surface was pre-conditioned by filling the well with a few drops of medium at 37°C for 15 min. The same amount of meticulously homogenized cell suspension (~15.000–30.000 cells/ml) was placed in the well and after 15–30 min incubation at 37°C the density and distribution of the cells were observed on an inverted microscope by phase-contrast. Medium (1.5–2 ml) was added by pouring on the wall to avoid disturbing settled cells. If necessary, one can rehomogenize cells in the well repeatedly using a syringe. Using ~30.000 cells/ml we obtained a working culture after around 24 h incubation, and with ~15.000–20.000 cells/ml after 48 h. Attached/flattened cells are in small groups distributed sparsely enough to orient and map their positions relatively to squares on the finder glass. (ii) The finder glass surface was pre-conditioned as above and 1.5–2 ml of a thoroughly homogenized cell suspension (~15.000–30.000 cells/ml) was poured starting from the well with a glass bottom. We agitated the cells in different directions and by circular motions during 5 min. After incubation at 37°C for 5–10 min the density and distribution of the cells was examined; this procedure shows less evenly distributed cells but good enough to find appropriate areas and mark position of individual cells and small groups. The results were significantly better if cells were repeatedly re-suspended using a 1 ml pipette.
(DOCX)

S6 Method Observations and imaging of living HeLa cells. We selected and examined single cells or groups by phase-contrast, Nomarski differential interference, and fluorescence at optical zooms of 100x and 200x (S6–S8 Figs). We registered timelapse z-series by SDCS using (i) Plan-Apochromat/63x/ with 1.4 numerical aperture and (ii) Plan-Apochromat/100x/1.46 objectives. Coordinates of appropriate COI were noted to enable their detection during time-lapse CM and imaging of the same cells after post-fixation immunolabeling. Exact cell location is especially important for CLEM analysis when the resin block must be trimmed to put the targeted cell in the central area of the pyramid and serial ultrathin sections. Before the cells were treated with AMD the nuclei of selected cells were examined by high magnification 3D LSM imaging by Nomarski and fluorescence. Histone H2B-GFP fluorescence was induced and recorded using 5% power of a 840 nm Chameleon Titanium/Sapphire biphoton pulsed laser. Fluorescent and Nomarski images were digitized simultaneously by Zeiss ZEN 2012 software. Volumes (512x512) were collected with 0.3 μm Z-steps using slow scanning mode and 1.3–1.5 digital zoom. Cells were briefly rinsed 3x during 5 min with PBS, immersed in fresh medium containing 0.05 μg/ml AMD, and the dish was immediately returned to the microscope plate holder and 4D images were collected as soon as possible. The cells were washed in PBS 3x during 5 min, prefixed for 10 min in 4% PAF in PBS, washed repeatedly and processed for anti-UBF immunolabeling, embedding in epoxy resin, and serial sectioning. Cultures for imaging by SDCS were previewed at 400x magnification and suitable cellular groups were identified in SDCS at low magnification and then used for high magnification 3D imaging of control stacks. Cultures were then rinsed 3x during 5 min with PBS and incubated in medium contained AMD for time-lapse imaging. Dishes were placed on the microscope stage and moved until COI were at their initial position. We collected images during 1–3 h, exciting GFP by 3% of Ar laser power at 491 nm using a BP530/50 emission filter with a 1 min interval between acquisitions. To distinguish the behavior of the ICC during nucleolar segregation time series were visualized in the form of 2D movies provided by ZEN 2011 or Quick Time software. Moreover, to visualize moving and coalescence of ICC clumps during the action of AMD the most significant points were extracted from the complete data-set and displayed one by one in chronological order as a gallery (Fig 6).
(DOCX)

S7 Method Post-fixation anti-UBF immunolabeling of He-La cells. Immediately after time-lapse imaging, AMD-treated samples were fixed in 4% PAF and rinsed (3x during 5 min) in PBS, permeabilized with 0.1% Triton X-100 in PBS during 5 min, and extensively washed in PBS. To block nonspecific binding they were incubated in 10% NGS (Jackson) in PBS during 30 min. After removing NGS the cells were covered by mouse anti-UBF/F-9 primary antibodies (1:50 in PBS) containing 1% NGS for 30 min and rinsed with PBS (3x5 min), then repeatedly incubated (15 min) in 10% NGS and for 30 min with biotinylated goat anti-mouse secondary antibodies (Jackson) (1:200 in PBS) containing 1% NGS. Secondary antibodies were detected by incubation for 15 min with streptavidin-Alexa568 (Invitrogen Molecular Probes, USA) (1:2000 in PBS) followed by washing in PBS overnight and examination under low magnification to locate previously imaged COI and to verify the quality of immunostaining. Volumes were recorded by LSM in simultaneous green-red regime using 1% of 840 nm Ar laser power, 488 nm excitation, and 561 nm emission. When post-fixed anti-UBF immunolabeled cells were imaged by SDCS, to excite GFP we used 3% laser power with 491 nm and a BP530/50 emission filter. SimultaneouslyAlexa568 was excited by 4% laser power at 591 nm using a BP 609/70 emission filter.
(DOCX)

S8 Method Preparation of double-labeled HeLa cells for CLEM. The area containing previously visualized COI was marked approximately on the opposite side of the finder grid, using a super fine permanent pen with dark color. Dehydration was in a graded series of ethanol-deionized water mixture, starting from 30% ethanol and then 50% (30 min), 70% (30 min), 80% (30 min), 90% (2 changes; each 15 min) and 96% (2x15 min). Impregnation was preceded by 2 changes of pure ethanol (30 min each). Impregnation was at room temperature in an ethanol-Embed 812 mixture containing Embed 812, DDSA, MNA and DMP30 in proportions to obtain hard embedding. The cells were immersed for at least 1.5–2 h at room temperature in 1:1 ethanol-Embed 812, then overnight at room temperature in ethanol-Embed 812 (1:2), then in Embed 812 (2 h each). The Embed 812 mixture was drained during 5–10 min to eliminate impregnation medium from the well and glass surface as much as possible. A droplet of resin was placed on the previously-marked ROI, drops of resin were used to glue the flat base of the resin cylinder over the ROI, and the assembly was transferred to a 60°C oven for 24 h. The resin containing flat embedded cells could be removed easily from the cover glass by careful bending and lifting of the cylinder. This technique is delicate because embedding media trapped in the gap under the resin cylinder contains air bubbles, so that the COI can be lost after polymerization. Bubbles could be slightly decreased by pre-incubation of the dish with a few drops of embedding medium to cover the cells for 1–2 h at 60°C. The best way to avoid bubbles is a two-step polymerization method, which is longer, but absolutely safe. The well was filled with embedding media and polymerized during 24 h at 60°C, and a droplet of resin was placed on the block over the marked area to attach the resin cylinder by a second polymerization for 24 h at 60°C. As a rule, after two-step embedding we needed to cut/scrape the resin block around the well edge as deeply as possible using a scalpel. The block carrying the COI flattened on its surface was detached from the finder glass as described above. If the coverslip broke while detaching, its fragments could be removed from the embedded cells by incubation in hydrofluoric acid (30–60 min in a plastic tube) to dissolve the glass [83]. After the resin had been detached from the glass the block was trimmed to eliminate resin and a pyramid was prepared with the COI in its center under the stereomicroscope of the ultramicrotome so that a global view of the finder grid could be seen at 0.7x magnification. Using a GEM single edge blade (EMS) we cut off excess resin to adjust the Ø14 mm block to the size of the cylinder. After identifying the ROI we continued trimming the block at 1.5-2x magnification to a ~ 1x1 mm pyramid with the selected cell approximately in its center and then at 3.5-4x magnification. For final trimming we used a fresh blade to leave a ~0.1–0.3 mm pyramid with the COI in its center. For sections collected on 1x2 mm formvar/luxfilm coated copper slot grids the pyramid had to be as narrow as possible, and for Maxtaform grids it was trimmed to fully cover the central hexagonal mesh to make sections tightly stretched around the COI close to the center of the section plane. During sectioning the trimmed face must be adjusted so that every point of the surface containing the COI is at the same distance from the knife edge to ensure the collection of sections beginning at the top surface of the block and penetrating the cell in the z direction to a depth of several tens of μM. Thus, the appropriate plane of interest containing nucleoli may be chosen during examination of serial sections in TEM and its depth noted. Then the corresponding depth may be readily calculated on 3D models constructed using LCM image stacks.
(DOCX)
