# Supplementary figures and images for: Nucleolar sub-compartments in motion during rRNA synthesis inhibition: Contraction of nucleolar condensed chromatin and gathering of fibrillar centers are concomitant
Source: PLoS One. 2017 Nov 30;12(11):e0187977. doi: 10.1371/journal.pone.0187977 (PMC5708645; doi:10.1371/journal.pone.0187977)

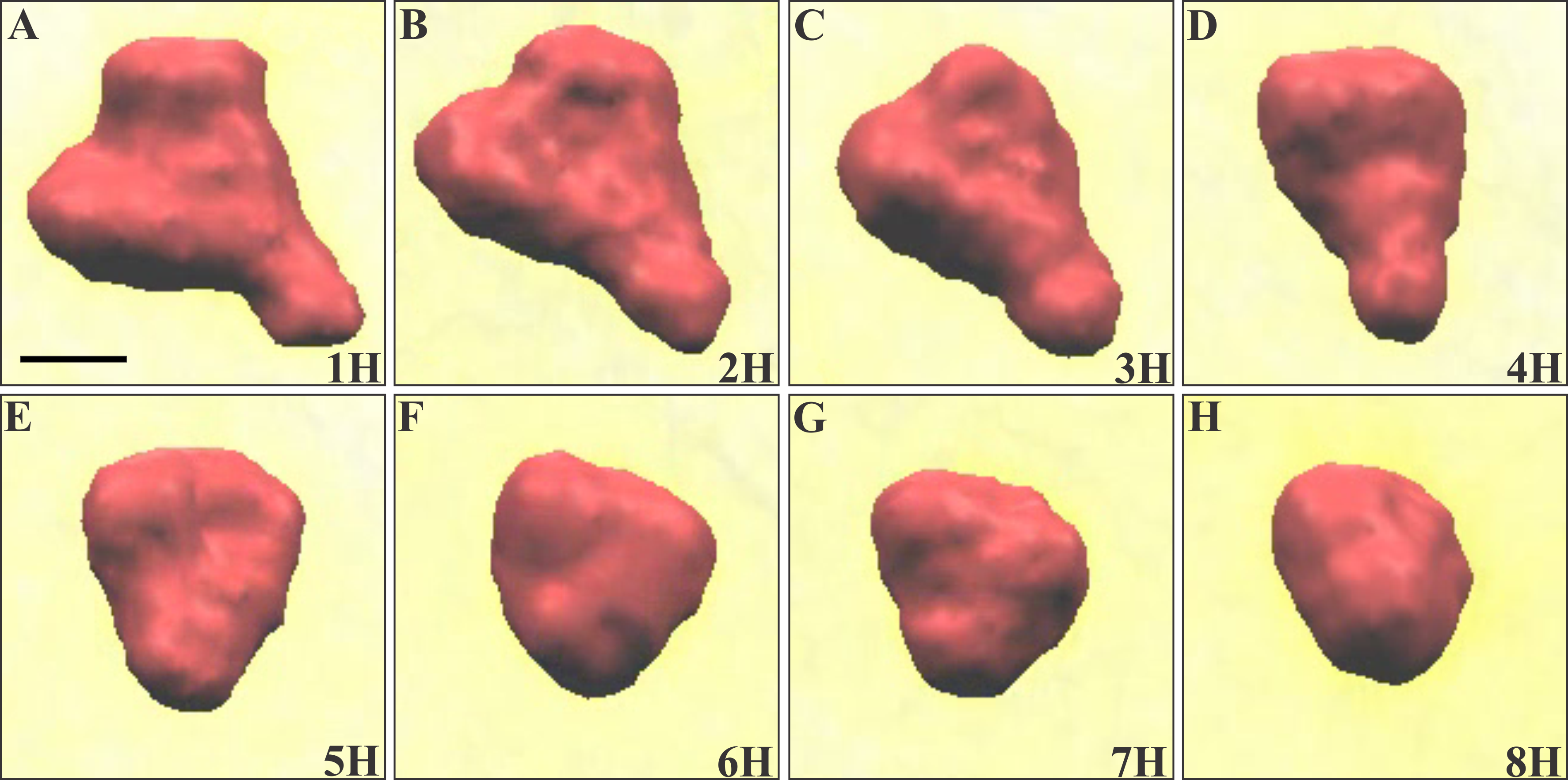

Supplement: S3 Fig — Corresponds to the cell presented on S1–S5 Movies. (A—H) Gallery of 3D reconstructions displaying nucleolar changes during the inhibition of rRNA synthesis. These reconstructions were performed using surface rendering at medium threshold to show nucleolar limits. Nucleoli, with an initial irregular shape, became spherical during inhibition. The scale bar represents 2 μm. (TIF) [file pone.0187977.s003.tif]

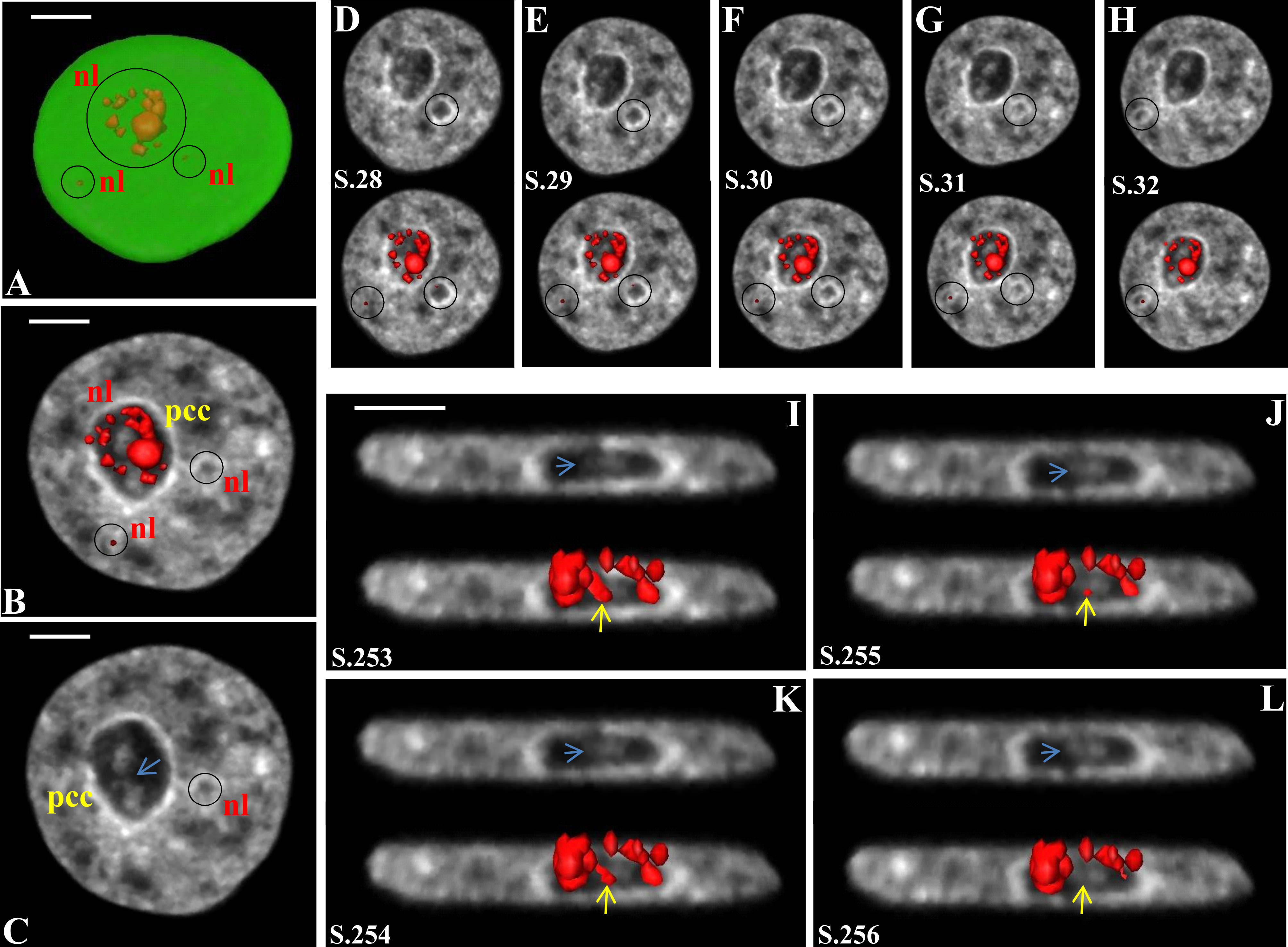

Supplement: S5 Fig — (A) 3D reconstruction of the nucleus (green) and immunolabeled UBF (red) in cells stably expressing H2B-GFP (transparent surface rendering). Black circles delineate the nucleoli (nl). (B, C) Two successive virtual sections (X/Y planes) revealing a strong PCC shell surrounding the nucleolus. Profound ICC strands (blue arrow) which are in a close structural link with UBF-positive NCs (S3B Fig) look like protrusions of PCC into the nucleolar space (S3C Fig). (D–L) Gallery of successive virtual sections cut in X/Y (S.28-32; S3D–S3H Fig) and X/Z (S.253-256; S3I–S3L Fig) planes shows the incorporation of ICC clumps with UBF-positive NCs on one side and ICC with PCC on another side. The close structural link between ICC (blue arrows) and UBF-positive NCs is obvious when imaged at different depths of cutting (yellow arrows). The scale bars represent 5 μm. (TIF) [file pone.0187977.s005.tif]

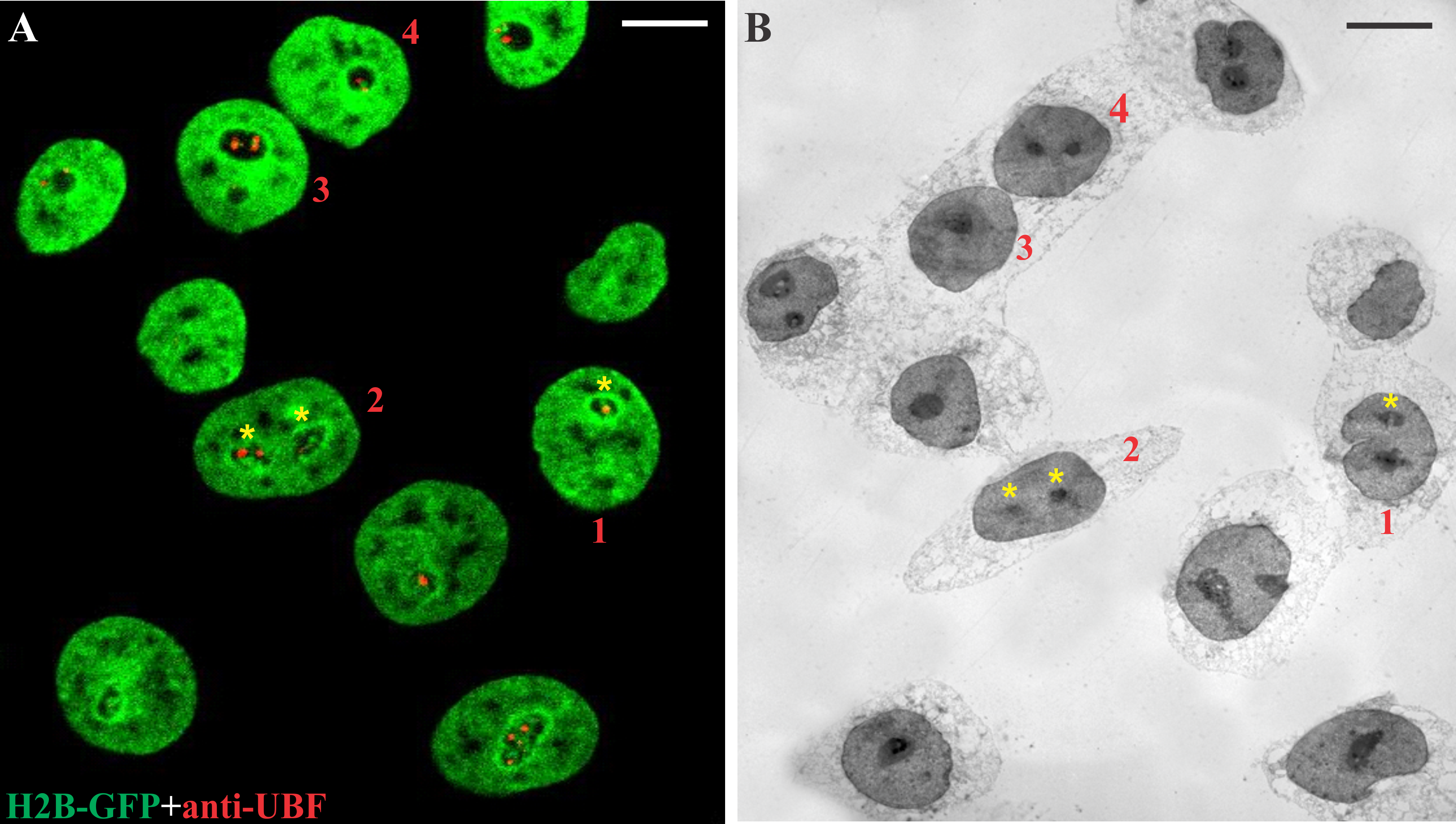

Supplement: S9 Fig — (A, B) After dehydration and plastic embedding, serial ultrathin sections of the same group of cells were imaged by electron microscopy. (A) H2B-GFP is green and UBF is red. All the nucleoli revealed UBF-positive red label. Note the structural continuity of ICC and UBF-positive spots. (B) On this section cells 1 to 4 are clearly identified (compare with S9A Fig). Nucleolar sub-components of cells N°1 and N°2 (marked by yellow stars) are shown in more detail in S10–S12 Figs. The scale bars represent 12 μm in (A); 16 μm in (B). (TIF) [file pone.0187977.s009.tif]

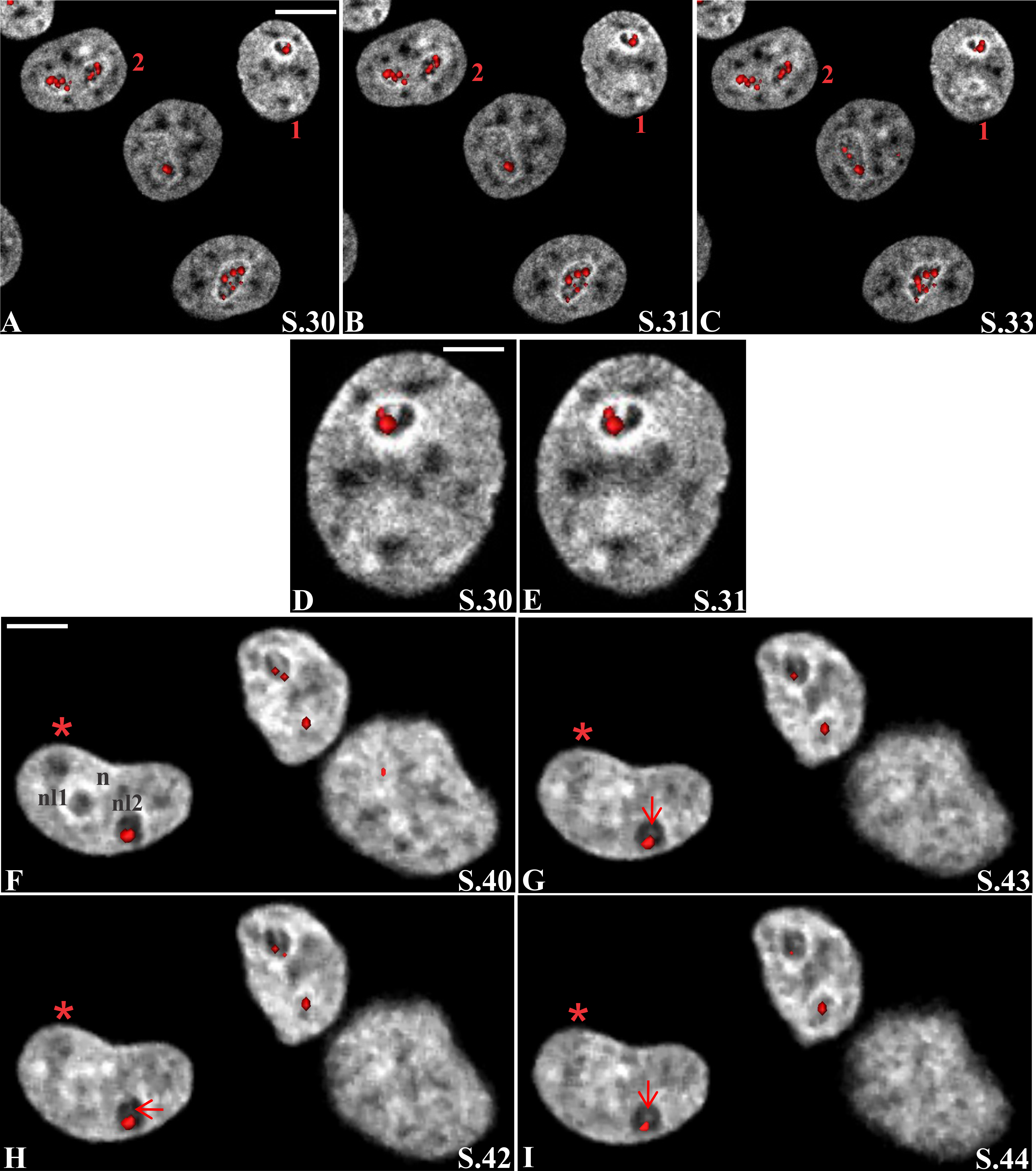

Supplement: S14 Fig — These images represent HeLa cells treated with AMD during 1 h. To correlate FCs and ICC with the structures fluorescently marked by anti-UBF antibody and H2B-GFP, we applied the confocal-transmission electron microscopy overlay technique (CTO) on the same cells (cells N°1 and N°2 in S9 Fig). To arrest the nucleolar transformation before segregation we fixed cells after 1 h or 1.5 h and performed anti-UBF immunolabeling. (A-C) Serial optical sections were combined with surface rendering for UBF-positive NCs (red) to follow the link between FCs and ICC in light and electron microscopy. (D, E) The nucleus of the cell N°1 selected for CLEM analysis at higher magnification. (F-I) Serial optical sections of the nucleus with a so-called ring-shaped nucleolus. Serial sections revealed one large UBF-positive FC in contact with prominent ICC clump. The scale bar represents: 12 μm in (A, C); 6 μm in (D, E); 8 μm in (F-I). (TIF) [file pone.0187977.s014.tif]

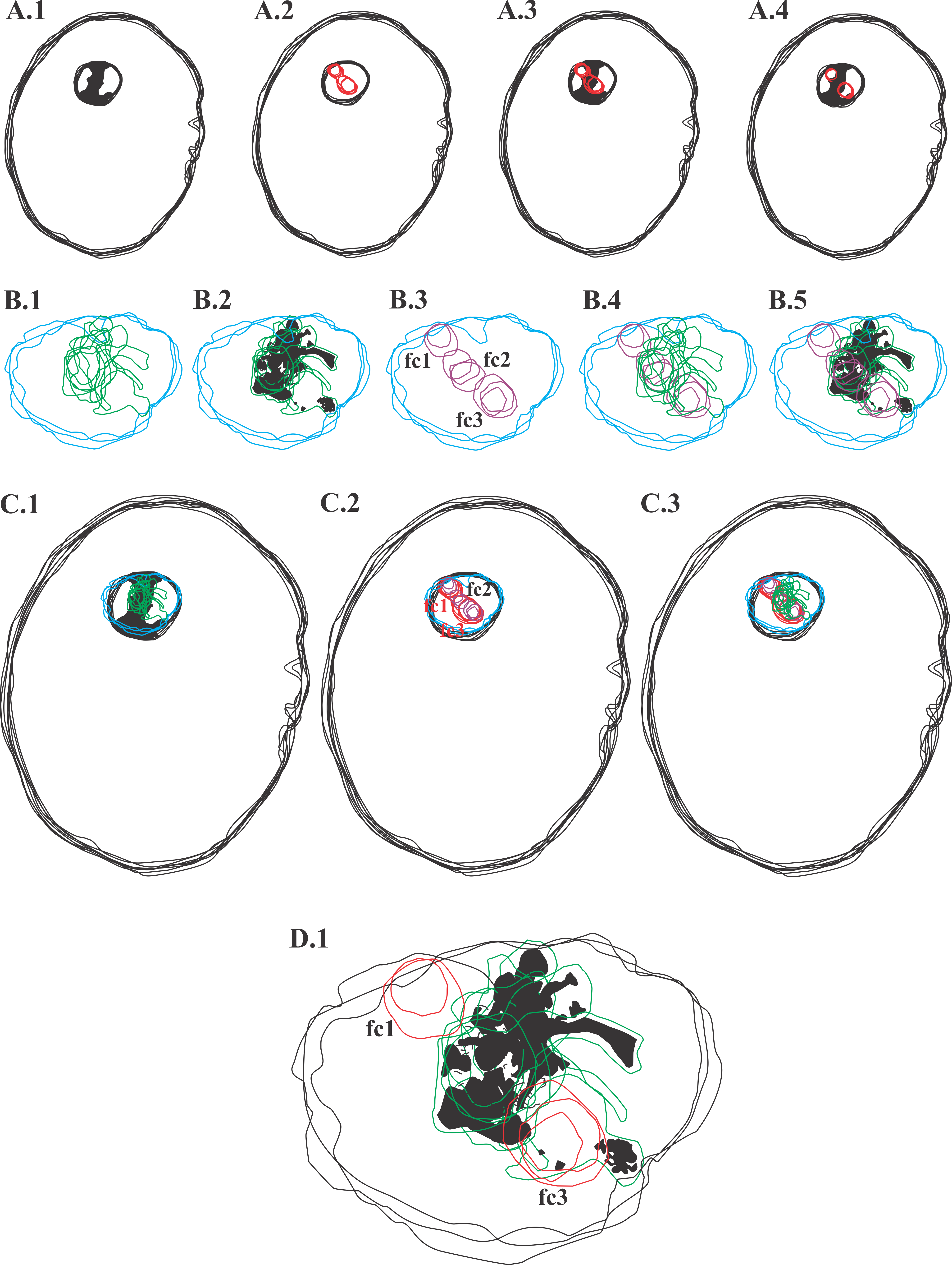

Supplement: S15 Fig — To perform CLEM, fluorescence images of the cell N°1 (S9 and S14A–S14E Figs) were rotated 90° to be properly aligned with the corresponding TEM images (S10 Fig). 3D reconstructions were performed by accurate matching as described in Materials and methods. (A1–4) Demonstration of structural relationship between condensed chromatin labeled by H2B-GFP (black) and anti-UBF antibody (red) using a successive colocalization approach. (A1) H2B-GFP forms a massive clump crossing the nucleolar volume (corresponds to S14D and S14E Fig). (A2) Due to lower resolution, only two UBF-positive NCs were distinguished at the LM level while in EM the corresponding cell reveals four FCs. (A3, A4) The structural link visible on the merged contours of H2B- and UBF-positive NCs repeats the picture observed on S14D and S14E Fig. (B1–5) 3D reconstructions performed by a successive colocalization of the contours taken from serial ultrathin sections of a corresponding nucleolus. (B1, 2) Colocalization of ICC and NVs (green): the massive ICC clump appears completely immersed in the complex network of NVs. (B3, 4) Colocalization of FCs (brown) with the network of NVs: FCs are always in contact with NVs. (B5) Colocalization of ICC, FCs and NVs (green): the unity of these NCs within the nucleolar volume is clear. (C1–3) The nuclear and nucleolar contours taken from the fluorescence images are marked in black and the nucleolar contours from serial ultrathin sections are marked in light blue. (C1) Localization of the fluorescent label (black) within the unit system of NVs (green) proves the identity of intra-nucleolar H2B-positive inclusions with ICC. (C2, C3) Perfect colocalization of the anti-UBF fluorescent label (red) with three FCs (brown) and NVs (green) definitively attributes UBF-positive structures to FCs. The 3D reconstruction in (D1) was constructed using the sections cut at the same depth along the z-axis as (A4). In both cases the massive ICC clump appears to be inserted between two FCs [file pone.0187977.s015.tif]

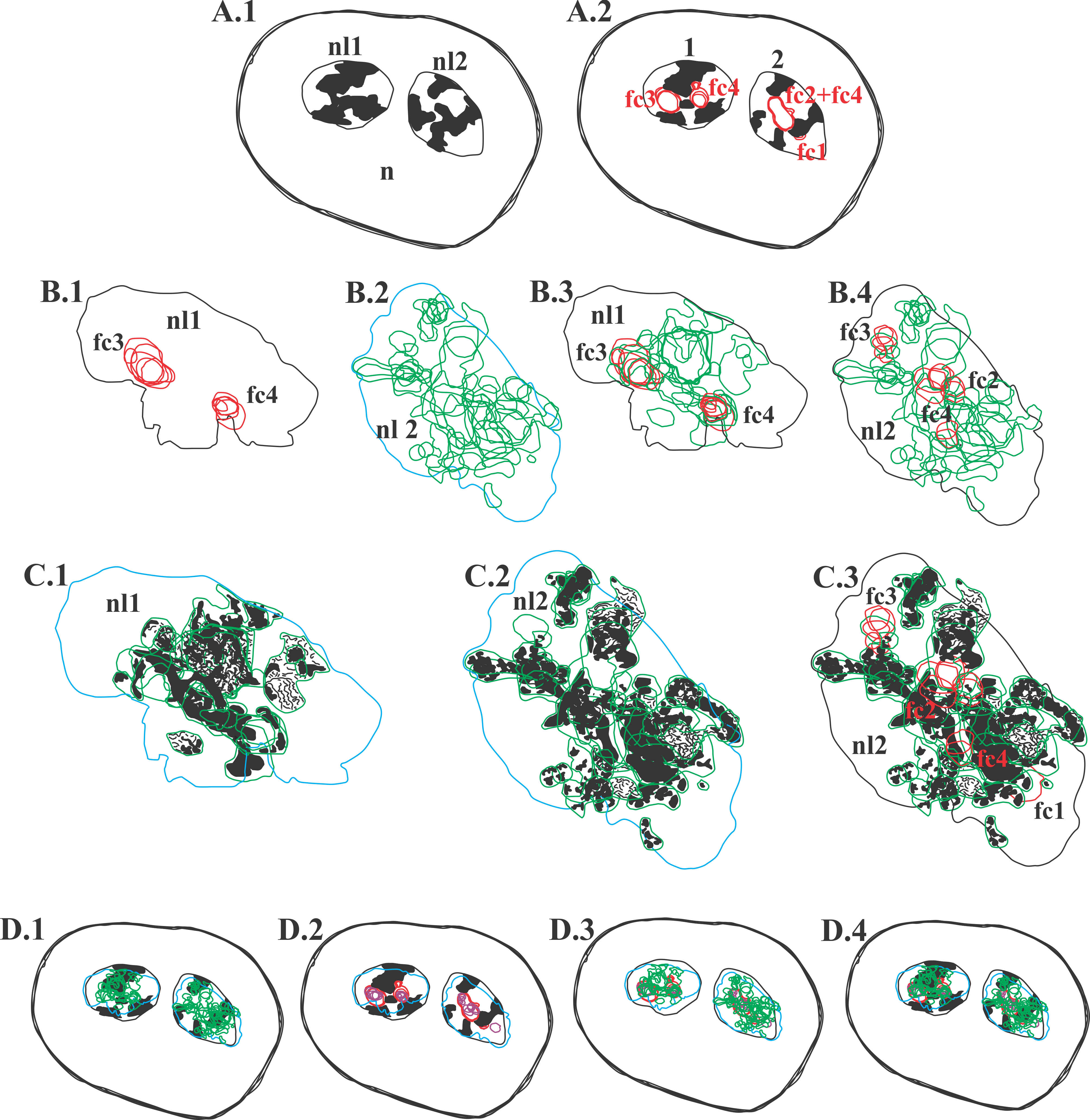

Supplement: S16 Fig — 3D reconstructions according to the nuclear and nucleolar contours outlined on fluorescence and EM images of two nucleoli (nl N°1 and nl N°2) inside the cell N°2 (corresponds to the S14A–S14C Fig). (A1, 2) Visualization of the structural link on the merged contours of the nucleus and NCs fluorescently labeled by H2B-GFP (black) and UBF (red), using a successive colocalization approach. (B1–4) 3D reconstructions obtained by the successive colocalization of contours taken from serial ultrathin sections of the nucleoli N°1 and N°2 (corresponds to S9 and S14A–S14C Figs). Colocalization of FCs (red) with the network of NVs (green): FCs are largely in contact with the vacuolar component of the nucleolus. (C1–3) Colocalization of ICC (black) and NVs (green) performed for nucleolus N°1 (C1) as well as ICC, FCs and NVs in nucleolus N°2 (C2, C3) in order to demonstrate the unity of these NCs. (D1–4) CTO of fluorescent and TEM images adjusted to the same magnification. The nuclear and nucleolar contours taken from fluorescence images are marked in black and the nucleolar contours on ultrathin sections in light blue. Colocalization of fluorescent H2B-GFP label (black) within the unit system of NVs (green) proves the identity of intra-nucleolar histone H2B-positive inclusions and ICC (D1). The colocalization of fluorescent anti-UBF label (red) with FCs (brown) and NVs (green) indicates the identity of UBF-positive structures and FCs (D2, D3). At the same time the colocalization of the fluorescent histone H2B-GFP and anti-UBF labels with ICC, FCs and NV (D4) demonstrates the unity of these NCs. (TIF) [file pone.0187977.s016.tif]

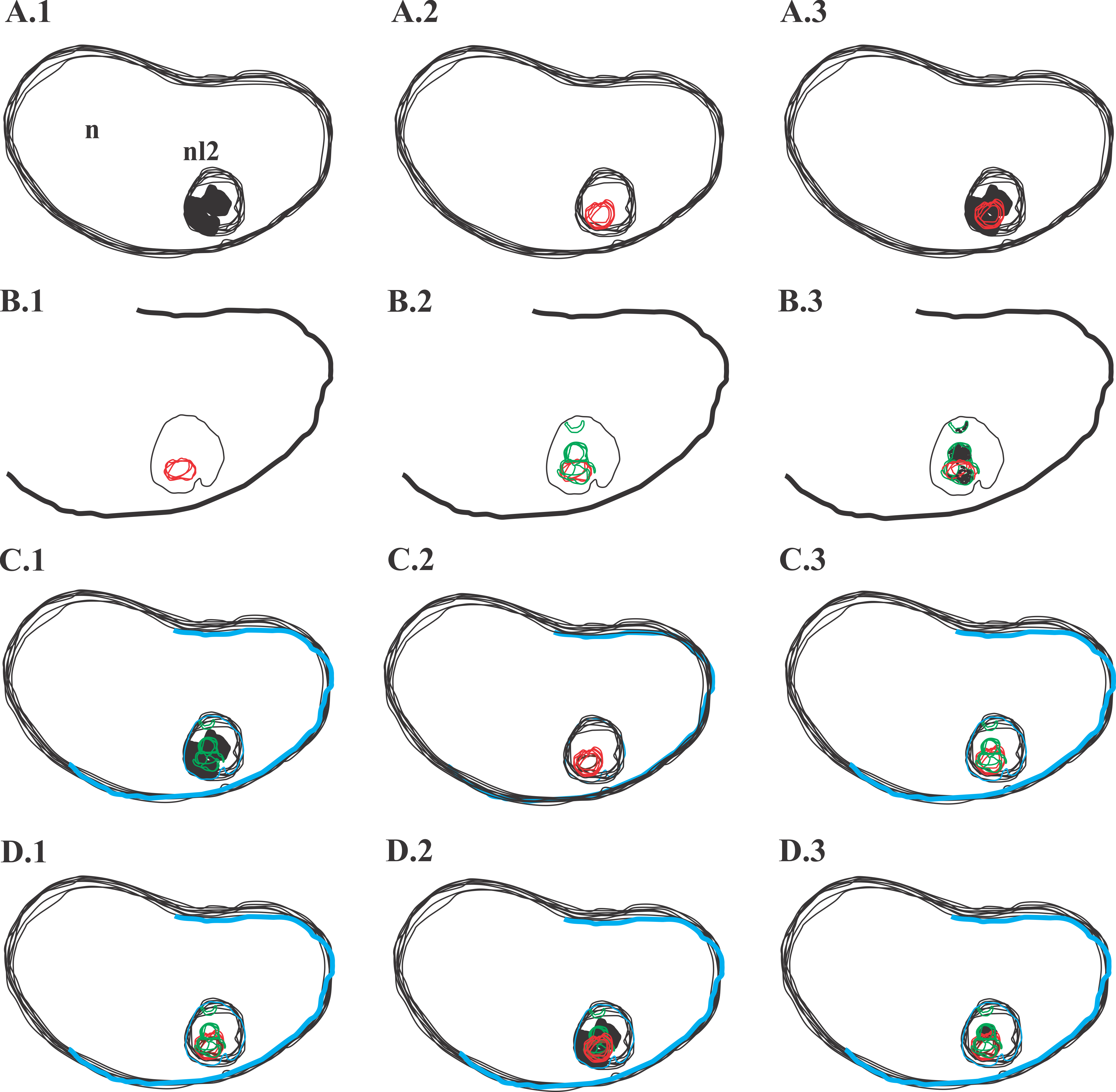

Supplement: S17 Fig — 3D reconstruction of the pre-segregated nucleolus in S13 and S14F–S14I Figs. Because of the presence of a large FC and a still profound NV these nucleolar modifications represent a suitable model to demonstrate the unity of the NCs. (A1–3) Successive colocalization approach to demonstrate the structural link between the massive intra-nucleolar block labeled by H2B-GFP (black) and UBF (red). (B1–3) EM colocalization of FCs (red), NV (green) and ICC (black): a massive ICC clump and FC are completely immersed in the NV (B3). (C1 –D3) merge of fluorescence and TEM images using a successive colocalization approach. The nuclear and nucleolar contours taken from the fluorescence images are marked by black, whereas identical contours outlined on the TEM images are light blue. Perfect colocalization of black and red fluorescence labels with ICC, FC and NV (green) proves the idea that these structures compose the unit system. (TIF) [file pone.0187977.s017.tif]
