# Supplementary material for: Nucleolar sub-compartments in motion during rRNA synthesis inhibition: Contraction of nucleolar condensed chromatin and gathering of fibrillar centers are concomitant
Source: PLoS One. 2017 Nov 30;12(11):e0187977. doi: 10.1371/journal.pone.0187977 (PMC5708645; doi:10.1371/journal.pone.0187977)
Supplement: S1 Method — To prevent formation of aggregates the cell suspension was agitated in different directions and by circular motion during a few min. Cells were incubated for 24 h at 37°C to reach 50–60% confluence, examined on an inverted microscope to select samples, rinsed with PBS, and immersed in fresh medium for 2–4 h. The transfection complex containing 4.8 μg cDNA and 7.2 μl Fugene-6 was prepared in 200 μl serum-free medium, incubated 15 min at room temperature, and poured over the cells growing in the last change of medium. (DOCX) [file pone.0187977.s027.docx]

**Method S1. Transient transfection/co-transfection** To prevent formation of aggregates the cell suspension was agitated in different directions and by circular motion during a few min. Cells were incubated for 24 h at 37ºC to reach 50-60% confluence, examined on an inverted microscope to select samples, rinsed with PBS, and immersed in fresh medium for 2-4 h. The transfection complex containing 4.8 μg cDNA and 7.2 μl Fugene-6 was prepared in 200 μl serum-free medium, incubated 15 min at room temperature, and poured over the cells growing in the last change of medium.
