# Supplementary material for: Nucleolar sub-compartments in motion during rRNA synthesis inhibition: Contraction of nucleolar condensed chromatin and gathering of fibrillar centers are concomitant
Source: PLoS One. 2017 Nov 30;12(11):e0187977. doi: 10.1371/journal.pone.0187977 (PMC5708645; doi:10.1371/journal.pone.0187977)
Supplement: S2 Method — Using a Bio-Rad MRC-1024ES/Olympus IX70 CM, GFP fluorescence was induced by excitation at 488 nm at 3–10% of Kr/Ar laser power in combination with VHS and Open filter blocks. Emission was recorded at 515 nm with an OG515 filter. In GFP/DsRed doubly-labelled preparations excitation was at 488 and 568 nm using 10% of laser power and T1 and T2 filter blocks, enabling simultaneous transmission of both bands and their recording through green and red channels (PMT2 and PMT1, respectively) with the following emission filters: for green 522DF35 (blocks 513 and 540 nm bands) and for red 605DF32 (blocks 589–621 nm). Images were acquired by Laser Sharp 3.2 software at zoom x4 corresponding to a pixel size of 0.080 μm, slow scanning speed mode (3 sec for each 512x512 image), and Kalman digital filtering (x3) to ameliorate signal/noise ratio. Phase contrast and fluorescence images were digitalized simultaneously. Consecutive optical sections (70–100 slices) were collected using 0.2 μm z-steps yielding stacks ~14–20 μm thick including entire KB cell nuclei. 3D localization of UBF and fibrillarin in fixed cells was reconstructed and visualized by Amira 5.5 software using two methods: (i) the “Isosurface” mode, displaying the exterior morphology (shape, number, and size) by surface rendering and (ii) the “Voltex” mode, representing interior organization by the rendering of the whole volume. 2D images were presented in “Maximum Intensity Projections” (MIP) mode after being processed using regular image-treatment and graphic software such as ImageJ, Corel Draw, and Photoshop. (DOCX) [file pone.0187977.s028.docx]

**Method S2. Observation and imaging of fixed KB cells.** Using a Bio-Rad MRC-1024ES/Olympus IX70 CM, GFP fluorescence was induced by excitation at 488 nm at 3-10% of Kr/Ar laser power in combination with VHS and Open filter blocks. Emission was recorded at 515 nm with an OG515 filter. In GFP/DsRed doubly-labelled preparations excitation was at 488 and 568 nm using 10% of laser power and T1 and T2 filter blocks, enabling simultaneous transmission of both bands and their recording through green and red channels (PMT2 and PMT1, respectively) with the following emission filters: for green 522DF35 (blocks 513 and 540 nm bands) and for red 605DF32 (blocks 589-621 nm). Images were acquired by Laser Sharp 3.2 software at zoom x4 corresponding to a pixel size of 0.080 μm, slow scanning speed mode (3 sec for each 512x512 image), and Kalman digital filtering (x3) to ameliorate signal/noise ratio. Phase contrast and fluorescence images were digitalized simultaneously. Consecutive optical sections (70-100 slices) were collected using 0.2 μm z-steps yielding stacks ~14-20 μm thick including entire KB cell nuclei. 3D localization of UBF and fibrillarin in fixed cells was reconstructed and visualized by Amira 5.5 software using two methods: (i) the “Isosurface” mode, displaying the exterior morphology (shape, number, and size) by surface rendering and (ii) the “Voltex” mode, representing interior organization by the rendering of the whole volume. 2D images were presented in “Maximum Intensity Projections” (MIP) mode after being processed using regular image-treatment and graphic software such as ImageJ, Corel Draw, and Photoshop.
