# Supplementary material for: Nucleolar sub-compartments in motion during rRNA synthesis inhibition: Contraction of nucleolar condensed chromatin and gathering of fibrillar centers are concomitant
Source: PLoS One. 2017 Nov 30;12(11):e0187977. doi: 10.1371/journal.pone.0187977 (PMC5708645; doi:10.1371/journal.pone.0187977)
Supplement: S3 Method — Using a Bio-Rad MRC-1024ES/Olympus IX70 CM, series of 60 optical slices with a z-step of 0.35 μm were recorded at zoom x3 and Kalman x2 digital filtering to ameliorate the signal/noise ratio. Recording of one Z-stack took 75 sec at fast scanning speed, including time for displacement along the z-axis. To prevent bleaching and/or photo damage we reduced illumination by around 20%, exciting GFP by 3% power of the Kr/Ar laser with B1/Open filter blocks. Emission was recorded as for fixed cells. Time-/z-series were recorded every 5 mins for 8 h. After 30 min the cells were perfused with AMD which was replaced by fresh medium after 2 h and collection was continued for 5.5 h. To demonstrate the general features in the behavior of the components containing fibrillarin and UBF we used simple visualization tools in the form of 2D movies provided by ImageJ or QuickTime. (DOCX) [file pone.0187977.s029.docx]

**Method S3. Observations and imaging of living KB cells.** Using a Bio-Rad MRC-1024ES/Olympus IX70 CM, series of 60 optical slices with a z-step of 0.35 μm were recorded at zoom x3 and Kalman x2 digital filtering to ameliorate the signal/noise ratio. Recording of one Z-stack took 75 sec at fast scanning speed, including time for displacement along the z-axis. To prevent bleaching and/or photo damage we reduced illumination by around 20%, exciting GFP by 3% power of the Kr/Ar laser with B1/Open filter blocks. Emission was recorded as for fixed cells. Time-/z-series were recorded every 5 mins for 8 h. After 30 min the cells were perfused with AMD which was replaced by fresh medium after 2 h and collection was continued for 5.5 h. To demonstrate the general features in the behavior of the components containing fibrillarin and UBF we used simple visualization tools in the form of 2D movies provided by ImageJ or QuickTime.
