# Supplementary material for: Nucleolar sub-compartments in motion during rRNA synthesis inhibition: Contraction of nucleolar condensed chromatin and gathering of fibrillar centers are concomitant
Source: PLoS One. 2017 Nov 30;12(11):e0187977. doi: 10.1371/journal.pone.0187977 (PMC5708645; doi:10.1371/journal.pone.0187977)
Supplement: S4 Method — Cells were briefly rinsed with PBS, fixed for 10 min in 4% paraformaldehyde in PBS adjusted to pH 7.2–7.4, rinsed repeatedly in PBS 3x5 min, and processed for CM and pre-embedding anti-GFP-fluoronanogold immuno-EM. Cells were incubated in 0.5% Triton X-100 in PBS for 5 min, in 10% normal goat serum (NGS) (Jackson, USA) in PBS for 30 min, and then with mouse monoclonal anti-human GFP antibody (Roche Diagnostics) (1:50 in PBS) for 30 min. The cells were rinsed repeatedly (15 min) in the above solution of NGS and incubated with goat anti-mouse biotinylated secondary antibody (Jackson) (1:100) for 30 min. Secondary antibodies were detected by a 15 min exposure to streptavidin-fluoronanogold conjugate (1:20 in PBS). Labeled cells were then postfixed in 1.6% glutaraldehyde and washed 3x10 min in PBS to remove glutaraldehyde completely. Importantly, when silver enhancer was used all procedures were at room temperature in light-tight boxes. Before silver enhancement cells were washed extensively (10x2 min) in deionized water. Silver enhancement was carried out at room temperature (about 20°C) for 7–9 min; the quality of staining is strongly temperature/time-dependent. To eliminate background staining enhancement was arrested by rapid immersion in ice-cold deionized water. The cells were thoroughly washed again (10x2 min) in deionized water and treated for 10 min in 5% aqueous sodium thiosulphate to quench residual metallic silver. The quality of labeling was controlled by phase contrast microscopy at 40x10 magnification with, removing the RS40 diaphragm. In properly stained cells the UBF positive nucleolar sites are clearly recognizable as dark brown, folded bead-like chains or relatively large distinct spots in sharp contrast against the pale yellow color of the nucleoplasm. (DOCX) [file pone.0187977.s030.docx]

**Method S4. Preparation of KB cells for anti-GFP immunolabeling and silver enhancement.** Cells were briefly rinsed with PBS, fixed for 10 min in 4% paraformaldehyde in PBS adjusted to pH 7.2-7.4, rinsed repeatedly in PBS 3x5 min, and processed for CM and pre-embedding anti-GFP-fluoronanogold immuno-EM. Cells were incubated in 0.5% Triton X-100 in PBS for 5 min, in 10% normal goat serum (NGS) (Jackson, USA) in PBS for 30 min, and then with mouse monoclonal anti-human GFP antibody (Roche Diagnostics) (1:50 in PBS) for 30 min. The cells were rinsed repeatedly (15 min) in the above solution of NGS and incubated with goat anti-mouse biotinylated secondary antibody (Jackson) (1:100) for 30 min. Secondary antibodies were detected by a 15 min exposure to streptavidin-fluoronanogold conjugate (1:20 in PBS). Labeled cells were then postfixed in 1.6% glutaraldehyde and washed 3x10 min in PBS to remove glutaraldehyde completely. Importantly, when silver enhancer was used all procedures were at room temperature in light-tight boxes. Before silver enhancement cells were washed extensively (10x2 min) in deionized water. Silver enhancement was carried out at room temperature (about 20°C) for 7-9 min; the quality of staining is strongly temperature/time-dependent. To eliminate background staining enhancement was arrested by rapid immersion in ice-cold deionized water. The cells were thoroughly washed again (10x2 min) in deionized water and treated for 10 min in 5% aqueous sodium thiosulphate to quench residual metallic silver. The quality of labeling was controlled by phase contrast microscopy at 40x10 magnification with, removing the RS40 diaphragm. In properly stained cells the UBF positive nucleolar sites are clearly recognizable as dark brown, folded bead-like chains or relatively large distinct spots in sharp contrast against the pale yellow color of the nucleoplasm.
