# Supplementary material for: Nucleolar sub-compartments in motion during rRNA synthesis inhibition: Contraction of nucleolar condensed chromatin and gathering of fibrillar centers are concomitant
Source: PLoS One. 2017 Nov 30;12(11):e0187977. doi: 10.1371/journal.pone.0187977 (PMC5708645; doi:10.1371/journal.pone.0187977)
Supplement: S5 Method — The concentration of cells must be adjusted to yield a culture sparse enough to facilitate localization of single cells or small groups. A satisfactory density on the finder grid was obtained if cells were seeded by the following 2 protocols (S6–S8 Figs). (i) The bottom glass surface was pre-conditioned by filling the well with a few drops of medium at 37°C for 15 min. The same amount of meticulously homogenized cell suspension (~15.000–30.000 cells/ml) was placed in the well and after 15–30 min incubation at 37°C the density and distribution of the cells were observed on an inverted microscope by phase-contrast. Medium (1.5–2 ml) was added by pouring on the wall to avoid disturbing settled cells. If necessary, one can rehomogenize cells in the well repeatedly using a syringe. Using ~30.000 cells/ml we obtained a working culture after around 24 h incubation, and with ~15.000–20.000 cells/ml after 48 h. Attached/flattened cells are in small groups distributed sparsely enough to orient and map their positions relatively to squares on the finder glass. (ii) The finder glass surface was pre-conditioned as above and 1.5–2 ml of a thoroughly homogenized cell suspension (~15.000–30.000 cells/ml) was poured starting from the well with a glass bottom. We agitated the cells in different directions and by circular motions during 5 min. After incubation at 37°C for 5–10 min the density and distribution of the cells was examined; this procedure shows less evenly distributed cells but good enough to find appropriate areas and mark position of individual cells and small groups. The results were significantly better if cells were repeatedly re-suspended using a 1 ml pipette. (DOCX) [file pone.0187977.s031.docx]

**Method S5. Preparation of He-La cells stably expressing fibrillarin-GFP and histone H2B-GFP for**

**time-lapse imaging.** The concentration of cells must be adjusted to yield a culture sparse enough to facilitate localization of single cells or small groups. A satisfactory density on the finder grid was obtained if cells were seeded by the following 2 protocols (S6 Fig – S8 Fig). (i) The bottom glass surface was pre-conditioned by filling the well with a few drops of medium at 37°C for 15 min. The same amount of meticulously homogenized cell suspension (~15.000-30.000 cells/ml) was placed in the well and after 15-30 min incubation at 37°C the density and distribution of the cells were observed on an inverted microscope by phase-contrast. Medium (1.5-2 ml) was added by pouring on the wall to avoid disturbing settled cells. If necessary, one can rehomogenize cells in the well repeatedly using a syringe. Using ~30.000 cells/ml we obtained a working culture after around 24 h incubation, and with ~15.000-20.000 cells/ml after 48 h. Attached/flattened cells are in small groups distributed sparsely enough to orient and map their positions relatively to squares on the finder glass. (ii) The finder glass surface was pre-conditioned as above and 1.5-2 ml of a thoroughly homogenized cell suspension (~15.000-30.000 cells/ml) was poured starting from the well with a glass bottom. We agitated the cells in different directions and by circular motions during 5 min. After incubation at 37°C for 5-10 min the density and distribution of the cells was examined; this procedure shows less evenly distributed cells but good enough to find appropriate areas and mark position of individual cells and small groups. The results were significantly better if cells were repeatedly re-suspended using a 1 ml pipette.
