# Supplementary material for: Nucleolar sub-compartments in motion during rRNA synthesis inhibition: Contraction of nucleolar condensed chromatin and gathering of fibrillar centers are concomitant
Source: PLoS One. 2017 Nov 30;12(11):e0187977. doi: 10.1371/journal.pone.0187977 (PMC5708645; doi:10.1371/journal.pone.0187977)
Supplement: S6 Method — We selected and examined single cells or groups by phase-contrast, Nomarski differential interference, and fluorescence at optical zooms of 100x and 200x (S6–S8 Figs). We registered timelapse z-series by SDCS using (i) Plan-Apochromat/63x/ with 1.4 numerical aperture and (ii) Plan-Apochromat/100x/1.46 objectives. Coordinates of appropriate COI were noted to enable their detection during time-lapse CM and imaging of the same cells after post-fixation immunolabeling. Exact cell location is especially important for CLEM analysis when the resin block must be trimmed to put the targeted cell in the central area of the pyramid and serial ultrathin sections. Before the cells were treated with AMD the nuclei of selected cells were examined by high magnification 3D LSM imaging by Nomarski and fluorescence. Histone H2B-GFP fluorescence was induced and recorded using 5% power of a 840 nm Chameleon Titanium/Sapphire biphoton pulsed laser. Fluorescent and Nomarski images were digitized simultaneously by Zeiss ZEN 2012 software. Volumes (512x512) were collected with 0.3 μm Z-steps using slow scanning mode and 1.3–1.5 digital zoom. Cells were briefly rinsed 3x during 5 min with PBS, immersed in fresh medium containing 0.05 μg/ml AMD, and the dish was immediately returned to the microscope plate holder and 4D images were collected as soon as possible. The cells were washed in PBS 3x during 5 min, prefixed for 10 min in 4% PAF in PBS, washed repeatedly and processed for anti-UBF immunolabeling, embedding in epoxy resin, and serial sectioning. Cultures for imaging by SDCS were previewed at 400x magnification and suitable cellular groups were identified in SDCS at low magnification and then used for high magnification 3D imaging of control stacks. Cultures were then rinsed 3x during 5 min with PBS and incubated in medium contained AMD for time-lapse imaging. Dishes were placed on the microscope stage and moved until COI were at their initial position. We collected images during 1–3 h, [file pone.0187977.s032.docx]

**Method S6. Observations and imaging of living HeLa cells.** We selected and examined single cells or groups by phase-contrast, Nomarski differential interference, and fluorescence at optical zooms of 100x and 200x (S6 Fig – S8 Fig). We registered timelapse z-series by SDCS using (i) Plan-Apochromat/63x/ with 1.4 numerical aperture and (ii) Plan-Apochromat/100x/1.46 objectives. Coordinates of appropriate COI were noted to enable their detection during time-lapse CM and imaging of the same cells after post-fixation immunolabeling. Exact cell location is especially important for CLEM analysis when the resin block must be trimmed to put the targeted cell in the central area of the pyramid and serial ultrathin sections. Before the cells were treated with AMD the nuclei of selected cells were examined by high magnification 3D LSM imaging by Nomarski and fluorescence. Histone H2B-GFP fluorescence was induced and recorded using 5% power of a 840 nm Chameleon Titanium/Sapphire biphoton pulsed laser. Fluorescent and Nomarski images were digitized simultaneously by Zeiss ZEN 2012 software. Volumes (512x512) were collected with 0.3 μm Z-steps using slow scanning mode and 1.3-1.5 digital zoom. Cells were briefly rinsed 3x during 5 min with PBS, immersed in fresh medium containing 0.05 μg/ml AMD, and the dish was immediately returned to the microscope plate holder and 4D images were collected as soon as possible. The cells were washed in PBS 3x during 5 min, prefixed for 10 min in 4% PAF in PBS, washed repeatedly and processed for anti-UBF immunolabeling, embedding in epoxy resin, and serial sectioning.

Cultures for imaging by SDCS were previewed at 400x magnification and suitable cellular groups were identified in SDCS at low magnification and then used for high magnification 3D imaging of control stacks. Cultures were then rinsed 3x during 5 min with PBS and incubated in medium contained AMD for time-lapse imaging. Dishes were placed on the microscope stage and moved until COI were at their initial position. We collected images during 1-3 h, exciting GFP by 3% of Ar laser power at 491 nm using a BP530/50 emission filter with a 1 min interval between acquisitions. To distinguish the behavior of the ICC during nucleolar segregation time series were visualized in the form of 2D movies provided by ZEN 2011 or Quick Time software. Moreover, to visualize moving and coalescence of ICC clumps during the action of AMD the most significant points were extracted from the complete data-set and displayed one by one in chronological order as a gallery (Fig 6).
