# Supplementary material for: Nucleolar sub-compartments in motion during rRNA synthesis inhibition: Contraction of nucleolar condensed chromatin and gathering of fibrillar centers are concomitant
Source: PLoS One. 2017 Nov 30;12(11):e0187977. doi: 10.1371/journal.pone.0187977 (PMC5708645; doi:10.1371/journal.pone.0187977)
Supplement: S7 Method — Immediately after time-lapse imaging, AMD-treated samples were fixed in 4% PAF and rinsed (3x during 5 min) in PBS, permeabilized with 0.1% Triton X-100 in PBS during 5 min, and extensively washed in PBS. To block nonspecific binding they were incubated in 10% NGS (Jackson) in PBS during 30 min. After removing NGS the cells were covered by mouse anti-UBF/F-9 primary antibodies (1:50 in PBS) containing 1% NGS for 30 min and rinsed with PBS (3x5 min), then repeatedly incubated (15 min) in 10% NGS and for 30 min with biotinylated goat anti-mouse secondary antibodies (Jackson) (1:200 in PBS) containing 1% NGS. Secondary antibodies were detected by incubation for 15 min with streptavidin-Alexa568 (Invitrogen Molecular Probes, USA) (1:2000 in PBS) followed by washing in PBS overnight and examination under low magnification to locate previously imaged COI and to verify the quality of immunostaining. Volumes were recorded by LSM in simultaneous green-red regime using 1% of 840 nm Ar laser power, 488 nm excitation, and 561 nm emission. When post-fixed anti-UBF immunolabeled cells were imaged by SDCS, to excite GFP we used 3% laser power with 491 nm and a BP530/50 emission filter. SimultaneouslyAlexa568 was excited by 4% laser power at 591 nm using a BP 609/70 emission filter. (DOCX) [file pone.0187977.s033.docx]

**Method S7. Post-fixation anti-UBF immunolabeling of He-La cells.** Immediately after time-lapse imaging, AMD-treated samples were fixed in 4% PAF and rinsed (3x during 5 min) in PBS, permeabilized with 0.1% Triton X-100 in PBS during 5 min, and extensively washed in PBS. To block nonspecific binding they were incubated in 10% NGS (Jackson) in PBS during 30 min. After removing NGS the cells were covered by mouse anti-UBF/F-9 primary antibodies (1:50 in PBS) containing 1% NGS for 30 min and rinsed with PBS (3x5 min), then repeatedly incubated (15 min) in 10% NGS and for 30 min with biotinylated goat anti-mouse secondary antibodies (Jackson) (1:200 in PBS) containing 1% NGS. Secondary antibodies were detected by incubation for 15 min with streptavidin-Alexa568 (Invitrogen Molecular Probes, USA) (1:2000 in PBS) followed by washing in PBS overnight and examination under low magnification to locate previously imaged COI and to verify the quality of immunostaining. Volumes were recorded by LSM in simultaneous green-red regime using 1% of 840 nm Ar laser power, 488 nm excitation, and 561 nm emission. When post-fixed anti-UBF immunolabeled cells were imaged by SDCS, to excite GFP we used 3% laser power with 491 nm and a BP530/50 emission filter. SimultaneouslyAlexa568 was excited by 4% laser power at 591 nm using a BP 609/70 emission filter.
