# Supplementary material for: Nucleolar sub-compartments in motion during rRNA synthesis inhibition: Contraction of nucleolar condensed chromatin and gathering of fibrillar centers are concomitant
Source: PLoS One. 2017 Nov 30;12(11):e0187977. doi: 10.1371/journal.pone.0187977 (PMC5708645; doi:10.1371/journal.pone.0187977)
Supplement: S8 Method — The area containing previously visualized COI was marked approximately on the opposite side of the finder grid, using a super fine permanent pen with dark color. Dehydration was in a graded series of ethanol-deionized water mixture, starting from 30% ethanol and then 50% (30 min), 70% (30 min), 80% (30 min), 90% (2 changes; each 15 min) and 96% (2x15 min). Impregnation was preceded by 2 changes of pure ethanol (30 min each). Impregnation was at room temperature in an ethanol-Embed 812 mixture containing Embed 812, DDSA, MNA and DMP30 in proportions to obtain hard embedding. The cells were immersed for at least 1.5–2 h at room temperature in 1:1 ethanol-Embed 812, then overnight at room temperature in ethanol-Embed 812 (1:2), then in Embed 812 (2 h each). The Embed 812 mixture was drained during 5–10 min to eliminate impregnation medium from the well and glass surface as much as possible. A droplet of resin was placed on the previously-marked ROI, drops of resin were used to glue the flat base of the resin cylinder over the ROI, and the assembly was transferred to a 60°C oven for 24 h. The resin containing flat embedded cells could be removed easily from the cover glass by careful bending and lifting of the cylinder. This technique is delicate because embedding media trapped in the gap under the resin cylinder contains air bubbles, so that the COI can be lost after polymerization. Bubbles could be slightly decreased by pre-incubation of the dish with a few drops of embedding medium to cover the cells for 1–2 h at 60°C. The best way to avoid bubbles is a two-step polymerization method, which is longer, but absolutely safe. The well was filled with embedding media and polymerized during 24 h at 60°C, and a droplet of resin was placed on the block over the marked area to attach the resin cylinder by a second polymerization for 24 h at 60°C. As a rule, after two-step embedding we needed to cut/scrape the resin block around the well edge as deeply as possible using a scal [file pone.0187977.s034.docx]

**Method S8. Preparation of double-labeled HeLa cells for CLEM.** The area containing previously visualized COI was marked approximately on the opposite side of the finder grid, using a super fine permanent pen with dark color. Dehydration was in a graded series of ethanol-deionized water mixture, starting from 30% ethanol and then 50% (30 min), 70% (30 min), 80% (30 min), 90% (2 changes; each 15 min) and 96% (2x15 min). Impregnation was preceded by 2 changes of pure ethanol (30 min each). Impregnation was at room temperature in an ethanol-Embed 812 mixture containing Embed 812, DDSA, MNA and DMP30 in proportions to obtain hard embedding. The cells were immersed for at least 1.5-2 h at room temperature in 1:1 ethanol-Embed 812, then overnight at room temperature in ethanol-Embed 812 (1:2), then in Embed 812 ( 2 h each). The Embed 812 mixture was drained during 5-10 min to eliminate impregnation medium from the well and glass surface as much as possible. A droplet of resin was placed on the previously-marked ROI, drops of resin were used to glue the flat base of the resin cylinder over the ROI, and the assembly was transferred to a 60ºC oven for 24 h. The resin containing flat embedded cells could be removed easily from the cover glass by careful bending and lifting of the cylinder.

This technique is delicate because embedding media trapped in the gap under the resin cylinder contains air bubbles, so that the COI can be lost after polymerization. Bubbles could be slightly decreased by pre-incubation of the dish with a few drops of embedding medium to cover the cells for 1-2 h at 60ºC. The best way to avoid bubbles is a two-step polymerization method, which is longer, but absolutely safe. The well was filled with embedding media and polymerized during 24 h at 60ºC, and a droplet of resin was placed on the block over the marked area to attach the resin cylinder by a second polymerization for 24 h at 60ºC. As a rule, after two-step embedding we needed to cut/scrape the resin block around the well edge as deeply as possible using a scalpel. The block carrying the COI flattened on its surface was detached from the finder glass as described above. If the coverslip broke while detaching, its fragments could be removed from the embedded cells by incubation in hydrofluoric acid (30-60 min in a plastic tube) to dissolve the glass [83]. After the resin had been detached from the glass the block was trimmed to eliminate resin and a pyramid was prepared with the COI in its center under the stereomicroscope of the ultramicrotome so that a global view of the finder grid could be seen at 0.7x magnification. Using a GEM single edge blade (EMS) we cut off excess resin to adjust the Ø14 mm block to the size of the cylinder. After identifying the ROI we continued trimming the block at 1.5-2x magnification to a ~ 1x1 mm pyramid with the selected cell approximately in its center and then at 3.5-4x magnification. For final trimming we used a fresh blade to leave a ~0.1-0.3 mm pyramid with the COI in its center. For sections collected on 1x2 mm formvar/luxfilm coated copper slot grids the pyramid had to be as narrow as possible, and for Maxtaform grids it was trimmed to fully cover the central hexagonal mesh to make sections tightly stretched around the COI close to the center of the section plane. During sectioning the trimmed face must be adjusted so that every point of the surface containing the COI is at the same distance from the knife edge to ensure the collection of sections beginning at the top surface of the block and penetrating the cell in the z direction to a depth of several tens of µM. Thus, the appropriate plane of interest containing nucleoli may be chosen during examination of serial sections in TEM and its depth noted. Then the corresponding depth may be readily calculated on 3D models constructed using LCM image stacks.
